# Human *Trypanosoma cruzi* infection in the Argentinean Chaco: risk factors and identification of households with infected children for treatment

**DOI:** 10.1186/s13071-024-06125-8

**Published:** 2024-01-29

**Authors:** Natalia P. Macchiaverna, Gustavo F. Enriquez, M Sol Gaspe, Lucía I. Rodríguez-Planes, Patricia R. Martinez, Ricardo E. Gürtler, M Victoria Cardinal

**Affiliations:** 1 Facultad de Ciencias Exactas y Naturales, Laboratorio de Eco-Epidemiología, Universidad de Buenos Aires, Ciudad Universitaria, Buenos Aires, Argentina; 2 Instituto de Ecología, Genética y Evolución de Buenos Aires, Consejo Nacional de Investigaciones Científicas y Técnicas, Ciudad Universitaria, Buenos Aires, Argentina; 3 Universidad Nacional de Tierra del Fuego, Instituto de Ciencias Polares, Ambiente y Recursos Naturales, Onas 450, 9410 Ushuaia, Argentina; 4 Administración de Parques Nacionales, Dirección Regional Patagonia Austral, Ushuaia, Argentina; 5 Hospital “Dr, Ezequiel Paulino Morante”, Avia Terai, Chaco, Argentina

**Keywords:** Chagas disease, *Trypanosoma cruzi*, Seroprevalence, Risk factor, Stratification, Indigenous

## Abstract

**Background:**

Chagas disease is a neglected tropical disease (NTD). Cost-effective strategies for large-scale implementation of diagnosis and etiological treatment are urgently needed to comply with NTD control goals. We determined the seroprevalence of *Trypanosoma cruzi* infection and associated risk factors in a well-defined rural population of Pampa del Indio municipality including creole and indigenous (Qom) households and developed two indices to identify houses harboring infected children.

**Methods:**

We serodiagnosed and administered a questionnaire to 1337 residents (48.2% of the listed population) in two sections of the municipality (named Areas II and IV) 6–9 years after deploying sustained vector control interventions. Multiple logistic regression models were used to evaluate the relationship between human infection and a priori selected predictors. Two risk indices were constructed based on environmental and serostatus variables, and we used spatial analysis to test whether households harboring *T. cruzi*-seropositive children were randomly distributed.

**Results:**

The global seroprevalence of *T. cruzi* infection was 24.8%. Human infection was positively and significantly associated with exposure time to triatomines, the household number of seropositive co-inhabitants, maternal seropositivity for *T. cruzi*, recent residence at the current house and the presence of suitable walls for triatomine colonization in the domicile. The pre-intervention mean annual force of infection (FOI) was 1.23 per 100 person-years. Creoles from Area IV exhibited the highest seroprevalence and FOI; Qom people from both areas displayed intermediate ones and creoles from Area II the lowest. Three hotspots of infected children were spatially associated with hotspots of triatomine abundance at baseline and persistent house infestation. No child born after vector control interventions was *T. cruzi* seropositive except for one putative transplacental case. Two simple risk indices (based on self-reported inhabiting an infested house and suitable walls for triatomines or maternal serostatus) identified 97.3–98.6% of the households with at least one *T. cruzi*-seropositive child.

**Conclusions:**

We showed strong heterogeneity in the seroprevalence of *T. cruzi* infection within and between ethnic groups inhabiting neighboring rural areas. Developed indices can be used for household risk stratification and to improve access of rural residents to serodiagnosis and treatment and may be easily transferred to primary healthcare personnel.

**Graphical Abstract:**

**Figure:**
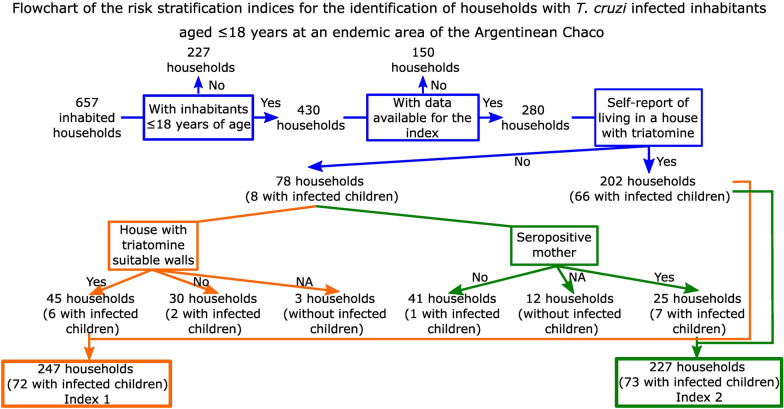

**Supplementary Information:**

The online version contains supplementary material available at 10.1186/s13071-024-06125-8.

## Background

Neglected tropical diseases (NTDs) disproportionately affect vulnerable resource-deprived populations from various ethnic groups often inhabiting rural areas [1]. These diseases are both consequence and causes of poverty, giving rise to “poverty traps” [2]. The global budget to control NTDs has historically been restricted. As part of the Millennium Development Goals, a renewed plan to eliminate NTDs has been launched, and Chagas disease is among the priorities for the Americas [3]. The Gran Chaco ecoregion, which mainly spreads over sections of Bolivia, Paraguay and Argentina, is a hotspot for several NTDs, including Chagas disease [4].

*Trypanosoma cruzi*, the etiological agent of Chagas disease, is transmitted by several routes: triatomine insects, which is the most important route in endemic regions [5]; transplacental or vertical route, the main source of new cases in non-endemic areas [6]; ingestion of food or beverages contaminated with infected triatomine feces [7], never reported in Argentina so far [8]; and blood transfusion and organ transplant, which have been blocked in the Americas by strict donor control [9]. Prevention of vector-borne transmission has mostly relied on insecticide spraying of houses since the 1950s [10–12]. Several regional initiatives have been launched to interrupt domestic transmission of *T. cruzi*. The degree of success has been heterogeneous throughout the continent, though a strong reduction in the geographic distribution of the main domiciliated vectors has been achieved [13]. However, insecticide resistance and rapid house reinfestation from untreated areas or houses have challenged control efforts in the Bolivian and Argentine Chaco regions[14].

A key step to diminish the burden of Chagas disease is to detect and treat *T. cruzi*-infected people, especially those < 18 years of age, who are eligible for treatment according to the current consensus [15]. Although treatment of chronic adults to prevent disease progression is still controversial, the new guidelines recommend treating women of childbearing age to block transplacental transmission and reduce the probability of acting as sources of *T. cruzi* to the vectors [16]. However, access to serodiagnosis and treatment has been historically poor, especially for the affected rural populations [17–19]. In remote rural areas, where access to the health system is scarce, strategies relying on house-to-house visits to perform blood collection for serodiagnosis were able to achieve high coverage proportions [17, 20–22].

Several studies have identified risk factors of human infection with *T. cruzi* at different levels: individual, household, social group or community, and landscape or region [23–31]. In general, duration of exposure, the presence and abundance of infected triatomines in domiciles, building materials of walls and roof, and presence and number of infected dogs and cats and of other infected human residents in the household (i.e. infected co-inhabitants) were associated with human *T. cruzi* infection in several scenarios, including the Gran Chaco region, where domestic transmission is mediated by *Triatoma infestans* (see [32] for a review). Risk factor analysis has been used to construct indices with the purpose of identifying individuals with higher infection risk. Some studies have relied on a few selected risk factors, such as infected vector densities, evidence of domestic infestation, self-reported inhabiting an infested house, building materials and *T. cruzi*-infected co-inhabitants, mother or father [33, 34]. Others used complex modeling with multiple sources of information and spatial analysis to identify households with higher risk of harboring an infected child [35]. To our knowledge, a simple-to-use, on-field risk index aimed to identify households with infected children, rather than infected individuals, has not yet been developed.

**Fig. 1:**
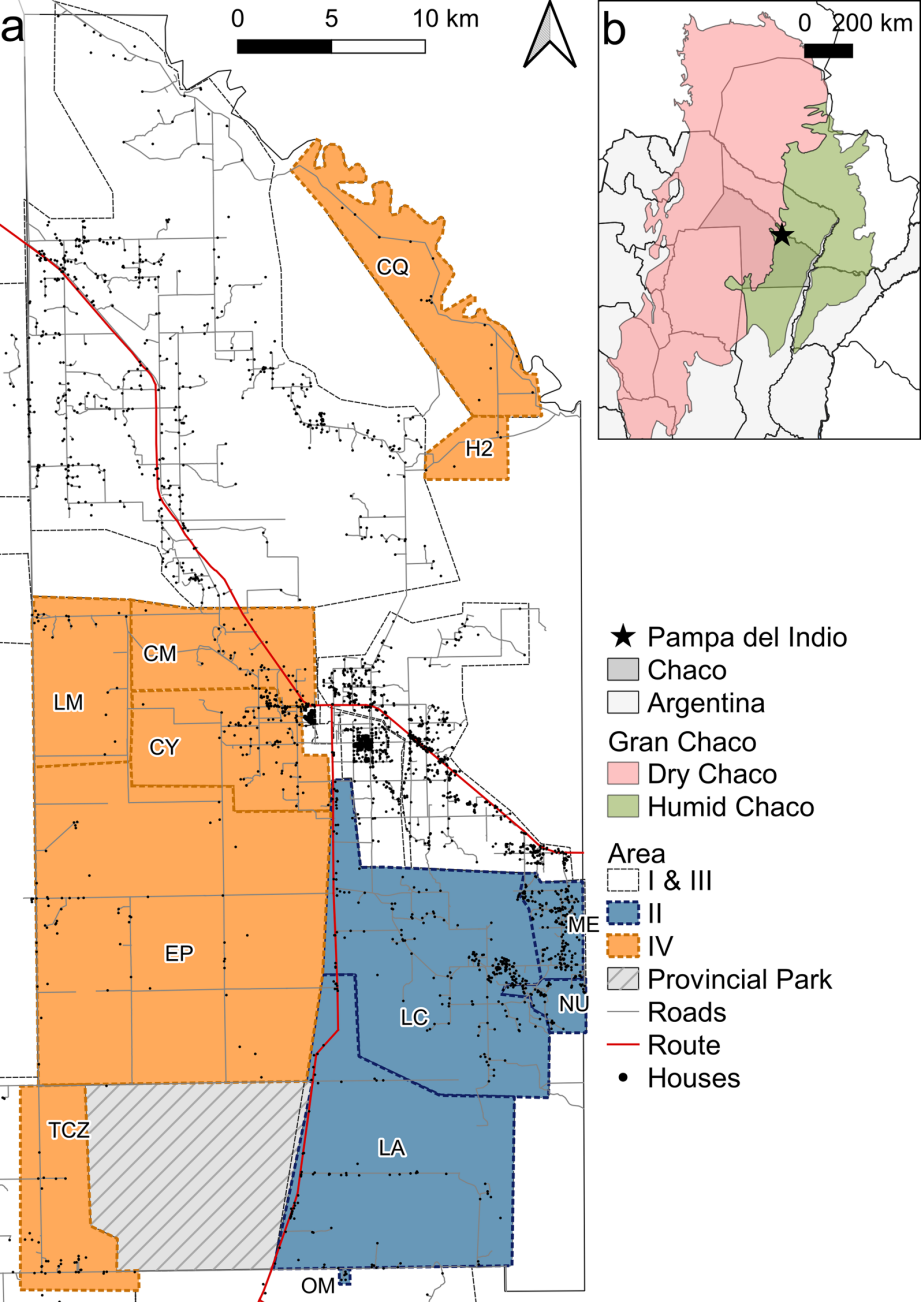
**a** Map of the study houses and communities from Areas II and IV shown as colored-shaded polygons. **b** Location of Pampa del Indio Municipality (star) within Chaco Province and the Gran Chaco ecoregion. CM, Colonia Mixta; CQ, Campo Cacique; CY, Campo Alemany; EP, Ex-Parque; H2, La Herradura; LA, Cancha Larga; LC, Lote Cuatro; LM, Las Muñecas; ME, Campo Medina; NU, Campo Nuevo; OM, Pampa Ombú; TCZ, Tacuruzal

This work is part of a long-term research program on the eco-epidemiology and control of Chagas disease launched in Pampa del Indio municipality (Argentine Chaco region) in 2007 [22, 36]. Two program goals were to eliminate *T. infestans* from all rural houses and to interrupt vector-borne transmission to humans by combining sustained vector control and surveillance with community participation, serodiagnosis and etiological treatment. The rural section of the municipality was divided into four areas for operational purposes (arbitrarily named Areas I–IV, Fig. 1). High levels of house infestation with *T. infestans* (range across areas: 14.4–41.4%) were recorded at baseline; a strong decline was achieved within 2 years of community-wide insecticide spraying and surveillance [22, 37–39]. *Trypanosoma cruzi* infection in *T. infestans*, dogs and cats was higher in Qom (the main ethnic group) households than in creole ones [36, 40], though infestation and human infection were not associated with household ethnicity when adjusted for other factors [41, 42]. The potential burden of Chagas disease was high as evinced by the levels of human seropositivity for *T. cruzi* recorded in extensive serosurveys covering Areas I and III [41]. Vector-borne transmission to humans was virtually interrupted shortly after the onset of the program, with no human incident cases detected among stable residents by 2016, and infection in only one child was attributed to vertical transmission [43].

In this study, we aimed to (i) determine the seroprevalence of human infection with *T. cruzi* in a well-defined rural population composed of creole and Qom people inhabiting Areas II and IV; (ii) identify risk factors associated with human infection; (iii) develop two simple risk indices to identify households harboring *T. cruzi*-infected children under 18 years of age; and (iv) test for spatial aggregation of houses harboring infected children. Given the strong decline of domestic infestation [22, 37–39] and the interruption of vector-borne transmission to humans [43], we hypothesized that children born after the onset of sustained vector control efforts would be seronegative for *T. cruzi*. In residents born before interventions, infection would be positively associated with the duration of exposure to triatomines and domicile infestation, belonging to the Qom ethnic group and the presence of *T. cruzi*-infected co-inhabitants. Infection is expected to be negatively related to the goat-equivalent index (a weighted average of livestock owned by the household in terms of goat biomass) and household educational level, which are surrogates of better socio-economic conditions [39, 44]. Households harboring infected children were expected to be spatially clustered because of the spatial aggregation of human infection [35, 43] and house infestation with *T. infestans* pre- and post-spraying [22, 37, 42, 45]. We also described socioeconomic characteristics of Area IV; those for Area II were reported elsewhere [37].

## Methods

### Study area

This study was conducted in the municipality of Pampa del Indio (26°2′0″S, 59°55′0″W), Libertador General San Martín Department, Chaco Province, Argentina. Pampa del Indio is located in the transition zone between the dry and humid Chaco (Fig. 1). According to the national census in 2010, 15,287 people lived in 3862 houses [46]. Fieldwork took place in Areas II and IV, which comprised 409 and 250 inhabited houses as of 2016, respectively, in the southern part of the municipality. The study area also comprised two small villages in the northern section (Campo Cacique, CQ, and La Herradura, H2), which had been intermittently inhabited because of occasional flooding; these houses were sprayed with insecticide along with those in Area IV.

Field interventions included a house-to-house survey and a community-wide insecticide spraying of the rural area of the municipality, which started at Area I in late 2007. These interventions were gradually scaled up to the rest of the municipality until achieving full coverage of rural sections in 2010. House infestation was periodically monitored thereafter. A full description and analyses of the effects of the interventions on house infestation, triatomine abundance and *T. cruzi* infection were described elsewhere [22, 36]. Briefly, in 2008, a systematic cross-sectional survey of house infestation was carried out in one of every three houses of Area II. Immediately after the baseline survey, house spraying with pyrethroid insecticide was implemented area-wide. In Area IV, the baseline vector survey was conducted in November 2009, May and November 2010 and was followed by community-wide insecticide spraying. Any house found infested with *T. infestans* during the surveillance phase was selectively sprayed with insecticides. In 2016 (i.e. 7–9 years after program onset), house infestation with *T. infestans* dropped below 1% [22].

### Study design

**Fig. 2:**
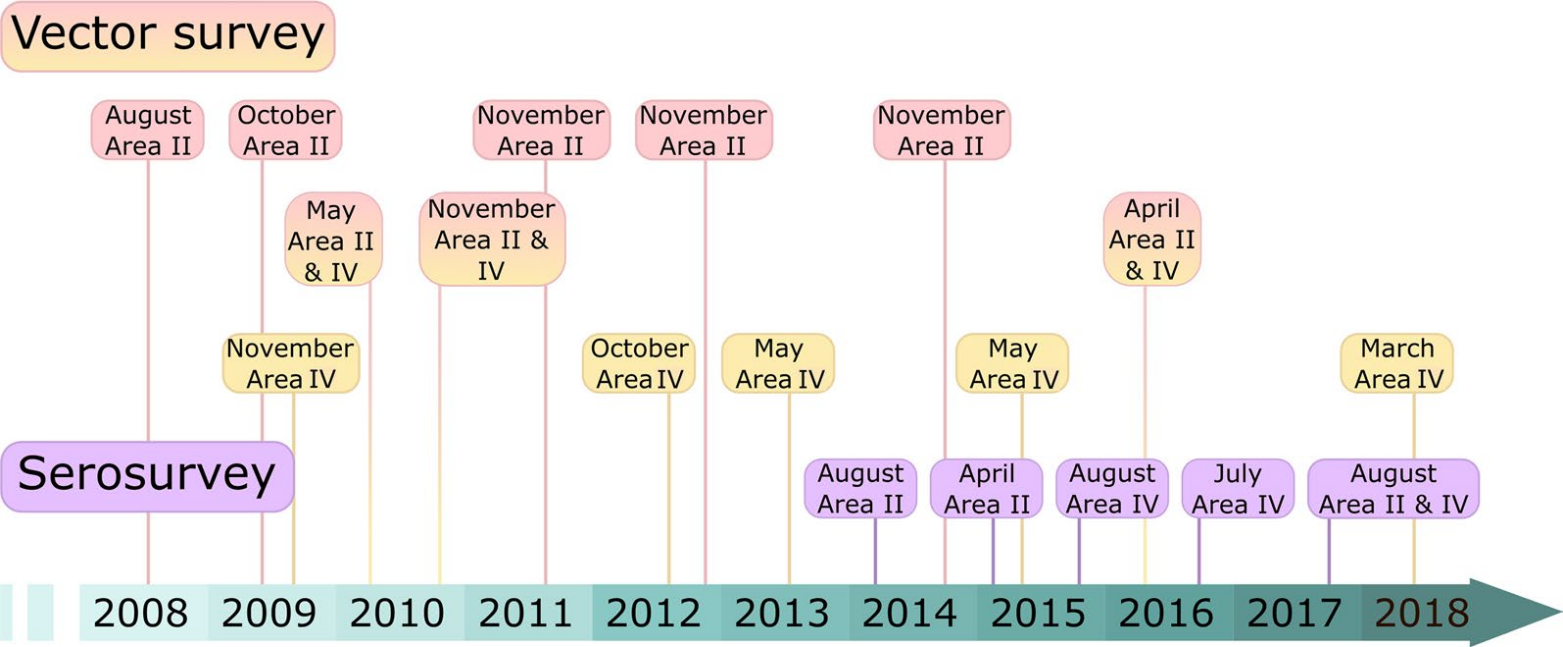
Timeline of key activities conducted at the study area. Pink and yellow boxes are vector surveys for Area II and IV, respectively. Purple boxes are the serosurveys at both areas

This study complies with the STROBE checklist (Additional file 1). Several cross-sectional serosurveys aiming at full coverage of rural residents were carried out in Areas II and IV over 2014–2017. We additionally collected socio-demographic information during house infestation surveys for risk factor analysis of human infection. A timeline of key activities is shown in Fig. 2.

### Vector and socio-demographic surveys

We conducted several house-to-house surveys to georreference each house, assess house infestation status by timed manual searches with a dislodging aerosol performed by vector control personnel [42] and record household environmental and sociodemographic features during 2008–2018 in Areas II and IV (Fig. 2). A house-to-house census of all rural households and residents was conducted in 2011 and 2014 for Area II and in 2015, 2016 and 2018 for Area IV. We recorded the full name of each household resident, age, relationship with the household head, permanent or occasional residence, alternative place of residence and whether they had previously been tested for *T. cruzi* infection and the outcome if known. We also scored each house as suitable for triatomine colonization or not based on the building materials of walls (mainly mud, thatch, sticks, brick with mud joints) or roofs (tarred-cardboard sheets, sticks or thatch, among others) and the presence of wall cracks as reported elsewhere [37]. Additionally, we recorded ethnic background for each household based on self-perception; whether they spoke Qomlataq language; participated in traditional Qom organizations or lived in ancestral indigenous territories. When in doubt, we checked with local health care personnel of Qom descent.

### Serosurvey

Five serosurveys were conducted in Areas II and IV in cooperation with the local hospital personnel during 2014–2017 (Fig. 2). Although all residents from Areas II and IV were eligible for serodiagnosis, we especially targeted children < 18 years of age. The exclusion criteria were: (i) children < 9 months of age since maternal antibodies to *T. cruzi* may still be detectable; (ii) children < 18 years of age without a parent or guardian available to provide written consent and (iii) adults unable to give consent.

We organized 10 informative meetings at different locations (i.e. local primary schools, healthcare posts, churches) to maximize participation across the study area. Invitations were sent through written notes to parents of schooled children, local radio interviews and primary healthcare agents. When necessary, local healthcare agents of Qom descent explained the information in Qomlataq language. After each meeting, a day and place were set to perform blood sample collection. At least one session was programed at each school or primary healthcare post in 2014 and 2015. House-to-house venipunctures were carried out in 2016 and 2017 to further increase diagnostic coverage. We asked all participants their full names, gender, ethnicity, mother’s name, maternal serostatus for *T. cruzi* (if known), actual and past residence place, name of the household head, if they recalled having inhabited an infested house and having received a blood transfusion, results of previous serodiagnosis for *T. cruzi* infection (if any) and whether the individuals that reported seropositivity have received etiological treatment for *T. cruzi* infection.

Prior to this study, 2064 people from Area I and III were serodiagnosed. The composite prevalence was 27.9% (95% CI = 26.0–29.9%). We calculated that the minimum sample size required to detect a prevalence of 27.9% with an error of 10%, including the finite population correction, was 757 inhabitants from Areas II and IV [47].

### Serodiagnosis

Up to 5 ml of blood was extracted by venipuncture from all participants aged ≥ 2 years. A retractile lancet was used for younger children, and the maximum blood volume obtained was 0.5 ml. Each serum sample was tested by at least two enzyme-linked immunosorbent assays (ELISA); one included *T. cruzi* epimastigote lysate (Chagatest, Wiener) and the other recombinant antigens (ELISA Rec V3.0, Wiener). Each sample was tested in duplicate by each ELISA test as previously reported [17]. A sample was considered seropositive for *T. cruzi* when it was reactive for two tests. Discordant samples were tested by indirect hemagglutination test (IHAT), ELISA and immunofluorescence antibody test (IFAT) at the National Institute of Parasitology “Dr. Mario Fatala Chabén” (ANLIS-Malbrán, Buenos Aires, Argentina) for final outcome.

Following national guidelines, all *T. cruzi*-seropositive people aged ≤ 18 years were referred for etiological treatment to the local hospital as reported elsewhere [17]. The local physicians also considered *T. cruzi*-seropositive people between 18 and 21 years of age and other seropositive adults who requested to be treated eligible for etiological treatment.

### Data analysis

Human infection data were merged with the georeferenced database that included house infestation and socioeconomic variables; the latter was available for Area II [37] and was compiled for Area IV in this study. Since the dates of intervention onset differed among communities (i.e. started in August 2008 for Area II and in November 2009 for CM, CY and LM, May 2010 for EP and November 2010 for CQ, H2 and TCZ), we estimated the time span (in years) each tested person was potentially exposed to triatomines by subtracting the time elapsed since the first insecticide spraying campaign at the individual’s community of residence to the age at examination [43]. We assumed that triatomine exposure occurring after control intervention was negligible compared with exposures before interventions based on the very low prevalence of domestic infestation after community-wide insecticide spraying [22, 36]. Therefore, all children born after the onset of interventions were assigned 0 years of exposure to *T. infestans*. To minimize the occurrence of missing values, whenever the maternal serostatus for *T. cruzi* infection was not reported we used the outcomes from our serosurveys; 318 participants reported maternal serostatus while we obtained serological information for 911 people.

We used Fisher’s exact tests and t-tests to compare proportions and means, respectively, and employed 95% Agresti-Coull confidence intervals or Wilson confidence intervals when the sample size was ≤ 40 (95% CI) [48]. Agreement between self-reported and observed serodiagnosis of *T. cruzi* infection was assessed using the kappa index.

### Risk factors

Generalized linear mixed-effects models (GLMMs) with a logit link function were used to evaluate the relationship between human infection and a priori selected predictors. We excluded 148 serodiagnosed people who had missing data in at least one variable from analysis. Analyses were performed with two different datasets to evaluate whether the risk factors differed between the complete population and children. Dataset 1 included all serodiagnosed inhabitants from Areas II and IV without missing data (*N* = 1189); dataset 2 (a subset from the first one) only included residents ≤ 18 years of age at baseline who had been born before the intervention program (*N* = 492). Based on previous evidence and preliminary analyses, the selected predictors were: duration of exposure to triatomines (in years, range: 0–78); household ethnicity (creole or Qom); area of residence (II or IV); the interaction between duration of exposure and ethnicity; self-reported inhabiting an infested house: whether the person (parents or guardians) recalled having ever inhabited an infested house; whether the mother was seropositive for *T. cruzi* infection (combining reported and tested serostatus as described above); number of *T. cruzi*-infected co-inhabitants detected by laboratory serodiagnosis performed in the current study (a three-level categorical variable: 0, 1–2 and 3 or more *T. cruzi*-infected co-inhabitants), whether the domicile had suitable walls for triatomines (yes or no); and time of residence at the current house up to 2016 (a three-level categorical variable: < 3 years, between 3–10 years and > 10 years). The household ID was included as a random factor. As maternal serostatus and the number of infected co-inhabitants were highly positively correlated (*P* < 0.001), for the first dataset, we included the number of infected co-inhabitants (which had no missing values), whereas for the second dataset, we included maternal serostatus.

Additional socio-demographic variables were not included in the multiple regression models because they exhibited high frequency of missing data or high correlation between variables. The goat-equivalent index quantifies the total number of livestock and poultry owned by the household in terms of goat biomass and is a household-level surrogate of wealth [45]. The goat-equivalent index was scaled by the biomass of 10 goats. The household educational level was computed as the mean number of schooling years attained by all house residents > 15 years old. Households with three or more occupants per room were under critical overcrowding [46]. The goat-equivalent index, educational level, critical overcrowding and time of residence were correlated; therefore, we only included time of residence in the models since it had fewer missing values. All numerical variables were centered.

Model 1 (dataset 1) was:

*T. cruzi* serostatus ~ duration of exposure + ethnicity + area + ethnicity $\times$ area + self-reported inhabiting an infested house + number of infected co-inhabitants + time of residence + suitable walls for triatomines ǁ (1 + Household ID).

Model 2 (dataset 2) was:

*T. cruzi* serostatus ~ duration of exposure + ethnicity + area + ethnicity $\times$ area + self-reported inhabiting an infested house + mother serostatus + time of residence + suitable walls for triatomines ǁ (1 + Household ID).

We used a multi-model inference approach to account for the uncertainty of model selection. Akaike’s information criterion (AIC) was used to identify the top-ranking models, i.e. the ones with a difference between the models AIC and the lowest AIC-scored model (ΔAIC) ≤ 2 AIC. The goodness of fit was assessed with the Hosmer-Lemeshow test and the area under the curve (AUC) of the receiver-operating characteristic (ROC) curve. The optimal threshold considered the value that minimized the total misclassification (i.e. false positives + false negatives). The H-index was used to assess classification performance penalized by the relative severity of one type of error over another: misclassifying a seropositive person as seronegative (false negative) was twice as costly as misclassifying a seronegative person as seropositive (false positive, i.e. severity ratio 0.5). All analyses were run in R 4.2.2 [49] using the packages “lme4” [50], "MuMIn” [51], "ResourceSelection" [52], “car" [53] and “hmeasure” [54].

Additionally, we constructed a simpler model to assess the association of *T. cruzi* infection and ethnicity, area and their interaction, accounting for exposure time, and the household ID. The data set for this analysis included all the participants (*n* = 1337) since all had record of age, ethnicity and house of residence (i.e. no lost cases due to missing data). This model was: *T. cruzi* serostatus ~ exposure time + ethnicity + area of residence + ethnicity x area of residence + (1|Household).

### Catalytic model

The mean annual force of infection (FOI) was estimated retrospectively using a catalytic model with a recovery rate set to 0 [55]. The incidence of infection is assumed to be constant over time and age; therefore, children born after the onset of vector control efforts were excluded. The model also assumes that no lag occurred between human infection and infectiousness, individual hosts are homogeneously exposed, and the observed prevalence is at equilibrium. The model was FOI = -ln(1-p_a_)/a, where “a” is the midpoint of the age class, and “p_a_” is the seropositive proportion for that class. For this analysis we used 13 age classes and estimated the average FOI for the overall population and for the four categories of ethnicity and area of residence. Additionally, we calculated an average FOI for children ≤ 18 years old at the onset of interventions with nine age classes.

### Index for risk stratification

We developed two simple indices to identify households with at least one seropositive resident ≤ 18 years of age, while minimizing the potential loss of households with infected children. We selected potential variables for the indices based on the outcomes of the risk factor analysis for individuals described above, the univariate and GLMM. For the index development variables, we considered that they were significantly associated with individual child infection for *T. cruzi*, or had high RI in the GLMM (RI ≥ 0.5), could be re-categorized to binary variables and could be imputed at the household level (excluding exposure time for example). Then, we performed univariate analyses (Chi-square test) with these variables. The eligible variables were: (i) self-reported inhabiting an infested house ever in their lifetime; (ii) suitable walls for triatomine colonization in domiciles; (iii) whether the mother of resident children was *T. cruzi*-seropositive; (iv) recent residence at the current house (< 3 years); (v) co-inhabiting with a *T. cruzi*-seropositive adult person (i.e. excluding infected co-inhabitants < 18 years old). Since access to serodiagnosis is limited in some Chagas disease-endemic regions, one index contained variables obtained from serodiagnosis, whereas the other did not to account for this limitation.

### Spatial analysis

To test whether the household number of *T. cruzi*-seropositive children ≤ 18 years of age at baseline was randomly distributed, we performed a global spatial analysis using the weighted K-function [56]. To evaluate the occurrence of aggregation of houses harboring seropositive children (i.e. hotspots), we performed local spatial analysis using the Getis Gi*(d) statistic [57]. Spatial analyses were implemented using the “spatstat” and “spdep” packages in R [58, 59]. The spatial analysis included 472 of 484 households with children ≤ 18-years old. We excluded six houses from CQ (a village with no spatial contiguity with the rest of the study area) and six houses that lacked georeferenced data.

## Results

### Population census

We registered 2793 inhabitants in both areas at the last census in 2018. Area II was the most populated one, with 1866 inhabitants residing in 380 houses, while we registered 927 people in 250 houses of Area IV (Additional file 2). The population was biased to males in both study areas (Chi-square test, χ^2^ = 31.34, df = 1, *P* < 0.001, male:female = 1.2 for Area II; χ^2^ = 26.11, df = 1, *P* < 0.001, male:female = 1.3 for Area IV). Mean age was not significantly different between areas (mean ± SD: 27.9 ± 21.4 and 27.2 ± 19.9 for Areas IV and II, respectively, t-test, *t*_(1423)_ = 0.80, *P* = 0.43). However, Area II had a significantly higher percentage of people < 18 years old (42.2%) than Area IV (38.0%) (Fisherʼs exact test, *P* = 0.03, OR = 1.19, 95% CI = 1.01–1.41, Additional file 3). In both areas, the mean age of creoles was higher than that of Qom residents (t-test, *t*_(573)_ = 8.80, *P* < 0.001 for Area II, t_(760)_ = 5.61, *P* < 0.001 for Area IV, Additional file 3).

### Socioeconomic and demographic features of Area IV

We enumerated 250 inhabited houses in the 503 km^2^ of Area IV in 2016. Population and house densities were 1.8 residents and 0.5 houses per km^2^, three times lower than in Area II (5.3 and 1.5, respectively). Area IV encompassed a significantly lower percentage of Qom households (34.4%) than Area II (70.9%) (Fisher’s exact test, *P* < 0.001, OR = 0.22, 95% CI = 0.15–0.31) (Additional file 2). For most of the socio-demographic variables analyzed in both areas, we observed more differences between ethnic groups than between areas, with a few exceptions (Additional file 3). Qom people in Area IV reported the shortest mean residence time. Qom people in Area II attained the lowest household educational level. The use of domestic insecticides was more frequent in both creole and Qom households from Area IV than Area II (Fisher’s exact test, *P* < 0.001, OR = 2.6, 95% CI = 1.5–4.7 for creole, *P* < 0.001, OR = 5.2, 95% CI = 1.9–16.3 for Qom). The median goat-equivalent index for Qom households was similar between areas (1.0, Q1-Q3 = 0.2–7.1 for Area II and 0.8, Q1-Q3 = 0.2–3.5 for Area IV). Creoles from Area IV (9.7, Q1-Q3 = 0.7–65.7, Additional file 2) reported a lower median goat-equivalent index than creoles from Area II (26.8, Q1-Q3 = 1.6–94.1), which is the lowest attained by creole households across the municipality (Kruskal-Wallis, χ^2^ = 14.64, df = 3, *P* = 0.002) [41, 45].

### Survey coverage

In total, 1446 people were examined for antibodies to *T. cruzi*. Of them, 1337 (93%) resided in the study area; the remaining were mostly from other rural or (peri)urban areas of Pampa del Indio municipality. Diagnostic coverage (overall, 47.9%) was significantly higher for women (52.8%, 95% CI = 50.1–55.7) than for men (43.8%, 95% CI = 41.4–46.3, Fisher’s exact test, *P* < 0.001, OR = 1.4, 95% CI = 1.2–1.7), implying an enriched sample of women compared with the census (Fisher’s exact test, *P* = 0.005, OR = 1.21, 95% CI = 1.06–1.38; Additional file 3). Diagnostic coverage for children ≤ 18 years of age was 55.4% (95% CI = 52.5–58.2); they were overrepresented in reference to the census (Fisher’s exact test, *P* < 0.001, OR = 1.3, 95% CI = 1.1–1.5). For women of childbearing age, the coverage was 56.5% (95% CI = 51.9–61.0). The lowest coverage was observed in children born after the onset of interventions (31.4%, 95% CI = 27.0–36.1), adults > 60 years of age (30.8%, 95% CI = 23.8–38.8) and young men aged 15–25 years (41.8%, 95% CI = 35.3–48.6). Overall, the examined population was enriched for Qom people (Fisher’s exact test, *P* < 0.001, OR = 1.4, 95% CI = 1.2–1.6) and was younger than the censused population (t-test, *t*_(2852)_ = 2.14, *P* = 0.03; Additional file 3). A total of 369 (53.7%) households had at least one participant in the serosurveys whereas 280 (57.9%) households had at least one person aged ≤ 18 years tested for *T. cruzi* antibodies.

### Self-reported and serodiagnosed *T. cruzi* infection or infestation status

A small fraction (13.2%) of the 1337 participants reported having a previous serodiagnosis for *T. cruzi* infection. Reported serostatus agreed with our serodiagnosis results in 82.4% of cases, i.e. a moderate agreement (κ = 0.53, 95% CI = 0.40–0.65). There were 31 incongruent cases. Eight of the 24 people who reported being seropositive but tested seronegative were children of whom we have records of having been treated for *T. cruzi* infection after testing seropositive; these eight cases probably indicated chemotherapeutic success rather than incongruent serodiagnostic reports. Additionally, 15 participants reported being seronegative but tested seropositive. We were not able to corroborate these diagnoses (no access to when, where or which serological tests were employed). Therefore, incidence of infection cannot be ruled out. We compared the reported and observed maternal serostatus for 186 participants and found a large fraction (84.0%) of congruent records, with a substantial agreement (κ = 0.67, 95% CI = 0.57–0.78). In total, 877 (65.6%) participants self-reported the presence of *T. infestans* at their residence in previous years; 362 (27.1%) people inhabited a house where *T. infestans* had been captured during the intervention program, and 76.8% reported exposure to triatomines.

### Seroprevalence of* T. cruzi* infection

**Fig. 3:**
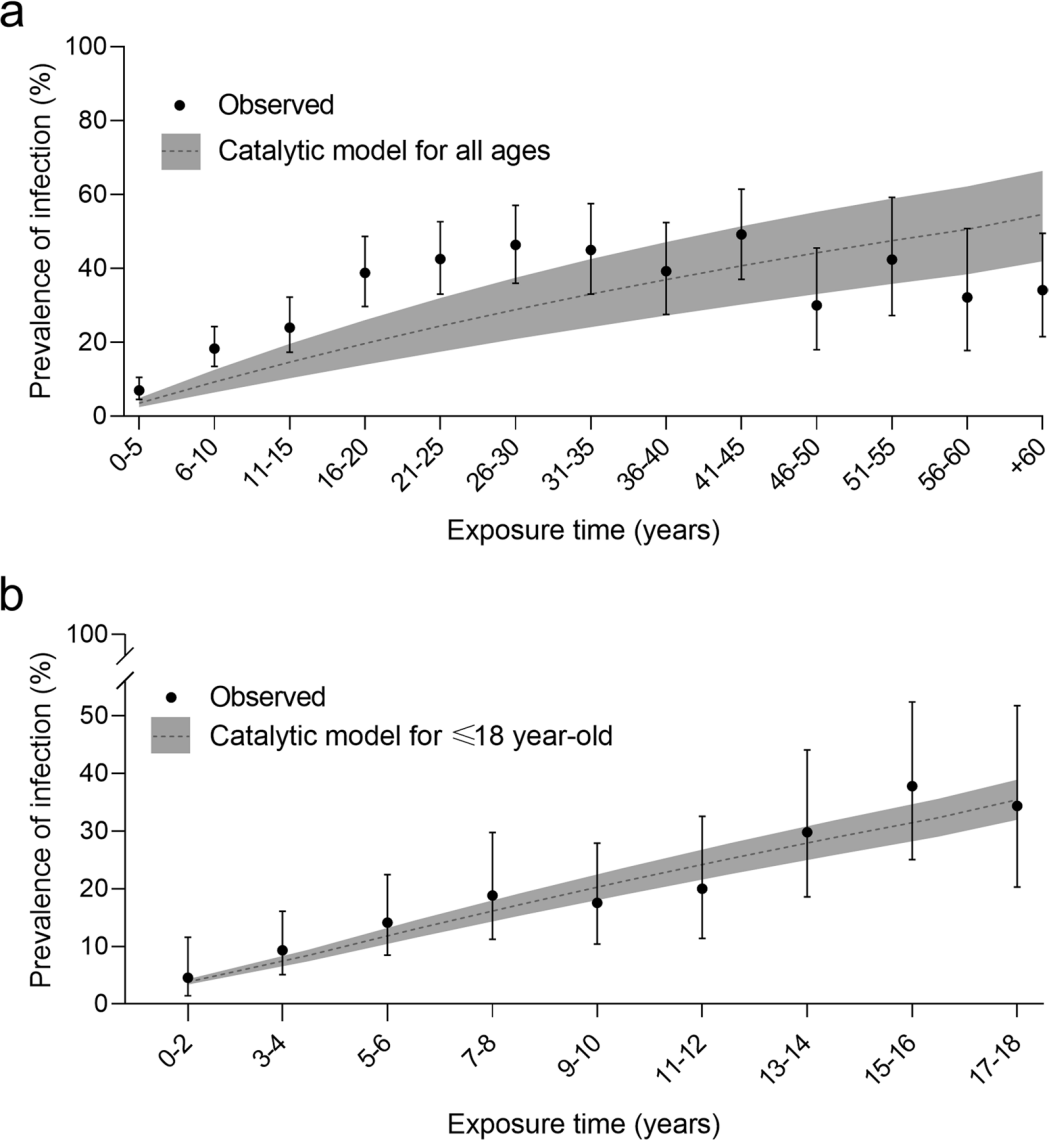
Age-specific seroprevalence of *Trypanosoma cruzi* infection in residents from Areas II and IV born before the onset of interventions, observed (dots) and 95% CI (whiskers) and predicted according to an irreversible catalytic model seroprevalence (dashed line and dark envelope). **a** For all ages and **b** for children ≤ 18 years old at the onset of interventions

The global seroprevalence of *T. cruzi* infection was 24.8% (95% CI = 22.6–27.2, n = 1337), and the age-adjusted prevalence was slightly higher (28.3%; 95% CI = 26.6–30.1). For people born pre-intervention, infection increased with age from 7% in children < 5 years to 39% in those aged 16–20 years, and thereafter fluctuated around 40% (Fig. 3a). Since there were 513 children ≤ 18 years of age without serodiagnosis and the observed seroprevalence of *T. cruzi* for this age group was 13.9%, we estimated that, on average, 71 (95% CI = 57–88) seropositive children probably remained without access to serodiagnosis and etiological treatment. Creoles from Area IV exhibited the highest seroprevalence (31.6%; 95% CI = 25.5–38.5); it was significantly higher than that of creoles (20.7%; 95% CI = 15.3–27.3) and Qom from Area II (24.1%; 95% CI = 21.1–27.1) but was similar to the seroprevalence of Qom people from Area IV (27.5%; 95% CI = 18.4–30.6; Table 1).

**Table 1:** Seroprevalence of *Trypanosoma cruzi* infection, odds ratio and estimated force of infection (FOI) according to household ethnicity and area of residence, Pampa del Indio, Chaco

Category	N	Mean exposure time ± SD	Seroprevalence (%, 95% CI)	FOI (95% CI)	Adjusted OR^a^ (95% CI)
Area II creole	174	29.5 ± 21.5	20.7 (15.3–27.3)	0.74 (0.30–1.23)	1
Area II Qom	775	16.3 ± 16.7	24.1 (21.3–27.3)	1.37 (0.94–1.90)	2.30 (1.27–4.18)
Area IV creole	196	20.5 ± 19.3	31.6 (25.5–38.5)	1.66 (1.13–2.35)	2.60 (1.29–5.25)
Area IV Qom	192	19.7 ± 17.1	27.5 (18.9–31.0)	1.16 (0.81–1.58)	2.02 (0.98–4.16)
Total	1337	18.6 ± 18.1	24.8 (22.2–26.8)	1.23 (0.88–1.66)	–

^a^ Model: *T. cruzi* serostatus ~ exposure time + ethnicity + area of residence + ethnicity x area of residence + (1|Household)

The estimated mean annual force of infection (FOI) was 1.23 per 100 person-years (95% CI: 0.88–1.66; n = 13 age groups) and for the children ≤ 18 years old at the onset of interventions was 2.52 per 100 person-years (95% CI: 2.21–2. 84; n = 9 age groups). For the catalytic model including all ages, the expected proportion of infected people differed from the observed ones in the age classes < 10 years and 16–25 years (Fig. 3a), while the expected values according to the catalytic model for children ≤ 18 years of age did not differ from the observed seroprevalence values (Fig. 3b). Creoles from Area IV and Qom people from Area II experienced a two-fold higher force of infection (1.66, 95% CI: 1.13–2.35; 1.37, 95% CI: 0.94–1.90, respectively) than creoles from Area II, who experienced the lowest force of infection (0.74, 95% CI: 0.30–1.23). Qom people inhabiting Area IV experienced an intermediate FOI (1.16, 95% CI: 0.81–1.58, Table 1).

Multiple logistic regression analysis of human infection revealed statistically significant effects of exposure time (OR = 1.05, 95% CI = 1.04–1.06, *P* < 0.001), operational area (Area IV OR = 2.6, 95% CI = 1.29–5.25, *P* = 0.008) and household ethnicity (Qom OR = 2.30, 95% CI = 1.27–4.18, *P* = 0.006) and a significant interaction term between operational area and household ethnicity (Area IV x Qom OR = 0.34, 95% CI = 0.14–0.83, *P* = 0.02). The adjusted OR of human infection for creoles from Area IV and Qom from Area II were 2.6 and 2.3 times greater than the creoles from Area II, respectively (Table 1).

We detected one (0.8%) *T. cruzi*-seropositive child out of 122 children born after the onset of interventions (Table 2). Her mother was also seropositive for *T. cruzi* and reported no previous etiological treatment. No triatomine was found at their house in any of the four surveys conducted during the follow-up period. Considering that most children born after the onset of interventions were apparently not exposed to infected *T. infestans* at their houses (Table 2) and 49 children born after intervention onset had an untreated *T. cruzi*-seropositive mother, the probability of vertical transmission was 2.0% (1 of 49, 95% CI = 0.04–10.7).

**Table 2:** Seroprevalence of *Trypanosoma cruzi* infection in children born after the onset of interventions, exposure to *Triatoma infestans* as determined by timed-manual collections over 2008–2016 and maternal seropositivity for *T. cruzi*, Areas II and IV, Pampa del Indio, Chaco, Argentina

Variable	N	% seropositive
House infestation with *T. infestans*^a^		
Yes	28	0
No	93	1.08
Domestic infestation with *T. infestans*^b^		
Yes	18	0
No	103	0.97
Presence of *T. cruzi-*infection in *T. infestans* as determined by OM or PCR^c^		
Infected	5	0
Not infected	17	0
No infection data	6	0
Maternal seropositivity		
Yes	49	2.04
No	71	0

a: *Triatoma infestans* collected at any site of the house

b: *Triatoma infestans* collected only in the domicile

c: Data from [36]

### Risk factors

In the first dataset, univariate analyses showed several variables that were positive and significantly associated with human *T. cruzi* infection: exposure time, being born before program onset, the number of seropositive co-inhabitants, maternal seropositivity, self-reported blood transfusion, domestic abundance of *T. infestans* at baseline, self-reported inhabiting an infested house, lower household educational climate, shorter time of residence, presence of suitable walls for triatomines and location of past residence (Additional file 4).

Using a GLMM approach, we identified seven top models for the first dataset (Model 1) and retrieved five top models for the second dataset (Model 2). In Model 1, the variables with higher relative importance (RI) were duration of exposure to triatomines, the number of infected co-inhabitants, time of residence at the current house and suitable walls for triatomines (Table 3). The risk of infection increased by 4% for each year of exposure; was five times higher for individuals with three or more infected co-inhabitants than for individuals with none; was higher for people living in houses with suitable walls for triatomines. People residing in their current house for > 3 years experienced a lower infection risk compared to more recent residents. Self-reported inhabiting an infested house (*P* = 0.15), ethnicity (*P* = 0.13) and area of residence (*P* = 0.15) were not significantly associated with human infection when adjusted for other predictors. The interaction between ethnicity and area was significant but had a low RI (0.35). The AUC of Model 1 was 0.77 and the H-index 0.21; the sensitivity of the averaged model was 72.0%, specificity 67.9% and explained deviance was 22.3%. The model exhibited a poor fit to the data according to the Hosmer-Lemeshow test (χ^2^ = 31.0, df = 8, *P* < 0.001). This is probably due to the high number of observations (> 1000), lack of saturation of the model or a non-linear relationship between variables.

**Table 3:** Generalized linear mixed model (logit link function) of human seropositivity for *Trypanosoma cruzi* infection for the total population (Model 1, *n* = 1189) and for children aged ≤ 18 years born before control interventions (Model 2, *n* = 492) from Areas II and IV, Pampa del Indio, Chaco. *RI* relative importance, *OR* Adjusted odds ratio; 95% CI 95% confidence interval

Variable	Model 1			Model 2		
	RI	OR (95% CI)	*P*	RI	OR (95% CI)	*P*
Duration of exposure (years)	1	1.04 (1.04–1.05)	< 0.001	1	1.25 (1.15–1.37)	< 0.001
Number of seropositive co-inhabitants	1			–	–	
0		1		–	–	–
1–2		1.20 (0.87–1.67)	0.26	–	–	–
3 or more		5.30 (3.44–8.17)	< 0.001	–	–	–
Maternal seropositivity	–	–	–	0.94		
No	-	–	–		1	
Yes	–	–	–		3.38 (1.37–8.34)	0.008
Time of residence (years)	1			0.8		
< 3		1			1	
3–10		0.43 (0.24–0.75)	0.003		0.13 (0.03–0.62)	0.01
> 10		0.48 (0.27–0.85)	0.01		0.16 (0.03–0.71)	0.02
Inhabiting a house with suitable walls for triatomines	1			0.6		
No		1			1	
Yes		1.47 (1.07–2.01)	0.02		2.29 (0.86–6.07)	0.1
Self-reported inhabiting an infested house	0.48			0.45		
No		1			1	
Yes		1.29 (0.92–1.82)	0.15		1.79 (0.73–4.37)	0.2
Ethnicity	0.43			0.38		
Creole					1	
Qom		1.49 (0.90–2.47)	0.13		1.67 (0.37–7.57)	0.5
Operational area	0.70			0.39		
II		1			1	
IV		1.63 (0.84–3.15)	0.15		1.38 (0.18–10.6)	0.8
Ethnicity × Area	0.35			0.14		
Creole × Area II		1			1	
Qom × Area IV		0.50 (0.25–1.00)	0.05		0.12 (0.01–1.16)	0.07

In Model 2, the variables with higher RI were duration of exposure to triatomines, maternal seropositivity to *T. cruzi*, time of residence and the presence of suitable walls for triatomines. Infection risk was three times higher for children born to a seropositive mother. A longer residence time at the same house was negatively associated with infection, as in Model 1. Duration of exposure to triatomines was positively associated with children infection. We observed a two-fold higher risk of infection for children living in houses with suitable walls for triatomines than for children living in houses without them, with marginal significance (*P* = 0.1). Ethnicity, area, their interaction and self-reported inhabiting an infested house were either not statistically significant or had low RI (Table 3). The random factor explained 32.8% of the deviance. The AUC was 0.96 and the H-index 0.68. The sensitivity of the averaged model was 91.3% and specificity 88.8%; the explained deviance was 60.8%, and the Hosmer-Lemeshow test was statistically significant (χ^2^ = 22.7, df = 8, *P* = 0.004).

### Identification of households with infected children

Following the risk factor analysis at individual level, we seek to identify the households harboring infected children. Based on the outcome of the univariate and GLMM risk factor analysis, the variables that significantly discriminated the 74 households with infected children were: self-reported residence in an infested house (Chi-square test, χ^2^ = 13.4, df = 1, *P* < 0.001), presence of suitable walls for triatomines (Chi-square test, χ^2^ = 4.1, df = 1, *P* = 0.04), maternal seropositivity (Chi-square test, χ^2^ = 25.5, df = 1, *P* < 0.001) and co-inhabiting with at least one seropositive adult person (Chi-square test, χ^2^ = 9.3, df = 1, *P* = 0.002) (Table 4).

**Table 4:** Variables evaluated for the development of risk indices to identify households with *Trypanosoma cruzi*-infected children ≤ 18 years old

Variable	N	TP	TN	FP	FN	Sensitivity	Specificity
Self-reported residence in an infested house	280	66	70	136	8	89.2	34.0
Maternal seropositivity	245	47	118	61	19	71.2	65.9
Co-inhabiting with a seropositive adult	280	45	125	81	29	60.8	60.7
Suitable walls for triatomines	259	49	82	109	19	72.1	42.9
Recent residence at the current house	272	12	174	26	60	16.7	87.0
Index 1	280	72	31	175	2	97.3	15.0
Index 2	280	73	52	154	1	98.6	25.2

*N* number of households with available data, *TP* true-positive, households with infected children correctly identified, *TN* true-negative households without infected children correctly identified, *FP* false-positive households without infected children incorrectly identified as harboring them. *FN* false-negative households with infected children incorrectly identified as not harboring them

A complete dataset to develop the indices was available for 280 households harboring children ≤ 18 years of age. For Index 1, we selected two variables with high sensitivity: self-reported residence in an infested house and the presence of suitable walls for triatomines. These variables identified 88% of the households (247/280) as having a high risk of harboring an infected child. Sensitivity and specificity were 97.3% and 15.0%, respectively (Table 4). The positive and negative predictive values were 29.1% and 93.9%, respectively. Index 2, which included self-reported residence in an infested house and maternal serostatus as predictors, identified 81% of the households as of high risk (227/280). Sensitivity was 98.6% and specificity 25.2%. The positive and negative predictive values were 32.2% and 98.1%, respectively.

Two households were false-negative cases for Index 1. In one household that reported no exposure to triatomines, we had collected domestic *T. infestans* at baseline. In the second household, the mother was seropositive, and this case was correctly classified by Index 2. Only one household with an infected child was incorrectly classified by Index 2. This household harbored two families: one had seronegative children and negative maternal serostatus, while the second family had a seropositive child but exposure to triatomines and maternal serostatus were unknown. As the household was classified according to the attributes of the first family, this led to a false-negative outcome.

In total, 204 households were excluded from previous analysis since no child ≤ 18 years of age had been tested serologically. Using the information available for Index 1 and 2 for those households excluded, we estimated that 91 and 37 households were at high risk of harboring infected children, respectively. According to the positive predictive values of each index, 26 and 11 households would harbor at least one infected child lost for diagnosis and treatment, respectively.

### Spatial analysis

**Fig. 4:**
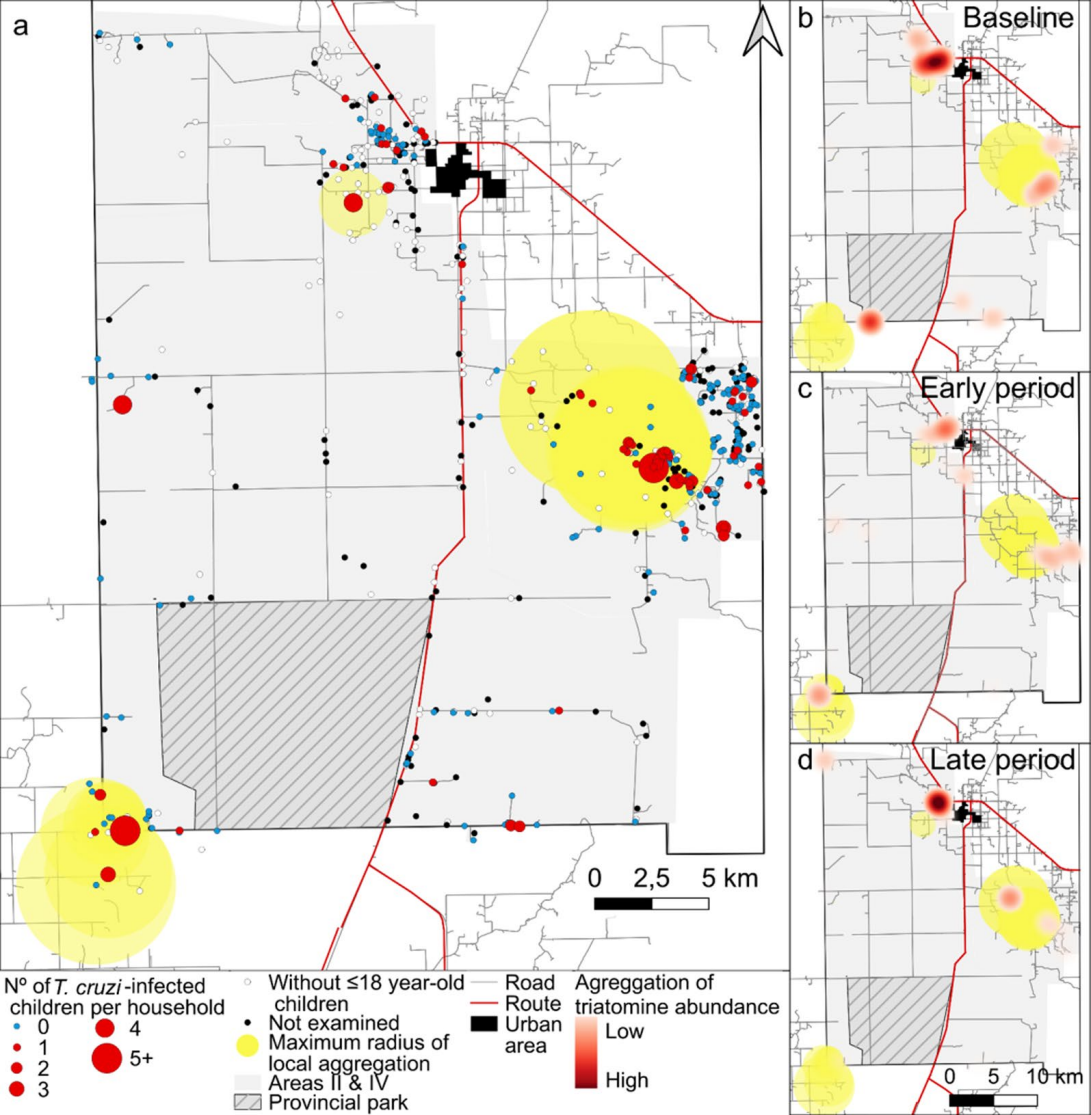
**a **Spatial distribution of households harboring *Trypanosoma cruzi*-seropositive children ≤ 18 years of age at baseline, Area II and IV, Pampa del Indio, Chaco. Yellow circles indicate local hotspots. Hotspots of households harboring *T. cruzi*-seropositive children and triatomine house abundance at **b** baseline, **c** early period of intervention and **d** late period of intervention as defined in [22]

Using global spatial analysis, we found that houses harboring infected children ≤ 18 years of age at baseline displayed no significant clustering for all study radii (14–7400 m, Additional file 5). Local spatial analysis of the number of infected children ≤ 18 years of age at baseline identified three significant hotspots encompassing 54 households within 100–4100 m radius. Most (80%) of these households occurred in a densely populated section of Lote Cuatro village in Area II (Fig. 4a). Two other hotspots were identified: one at the southwest corner of the municipality and the other close to urban Pampa del Indio. The three hotspots of houses harboring infected children overlapped with or were close to previously detected hotspots of *T. infestans* abundance at three interventions periods (Fig. 4b-d) [22].

## Discussion

The global age-adjusted prevalence of infection with *T. cruzi* of Areas II and IV inhabitants remained high at 28.7%, 6–9 years after the onset of sustained vector control interventions in Pampa del Indio. Infection was positively and significantly associated with duration of exposure to triatomines, the number of seropositive co-inhabitants, maternal serostatus, presence of suitable walls for triatomines and recent residence at the current house. The interaction between ethnicity and operational area had weak effects when adjusted for other predictors in the full models. The sustained vector control actions apparently prevented the occurrence of new infections since no tested child born after program onset was seropositive, except one who may have acquired *T. cruzi* from the seropositive mother as recorded elsewhere [43]. The two risk indices showed high sensitivity (97.3–98.6%) at the expense of low specificity (15.0–25.2%), and had nearly perfect negative predictive values (93.9–98.1%) when applied to the identification of households harboring infected children ≤ 18 years of age. Additionally, we identified three hotspots of houses harboring infected children that overlapped with hotspots of triatomine abundance at baseline and persistent house infestation [22, 37].

The global human seroprevalence of *T. cruzi* in Areas II and IV was lower than in Areas I (39.8%) and similar to that registered in Area III (29.0%) [41]. These studies were conducted 3 and 4–6 years after the onset of the interventions, respectively [41], whereas the current study was conducted 6–9 years after sustained vector control actions. Operational areas also differed in the observed baseline household *T. infestans*-infestation prevalence and abundance [22]. Other locations of the Gran Chaco with active vector-borne transmission and lack of sustained vector control showed higher prevalence of infection [24, 60–68]. Similarly, the pre-intervention force of infection (FOI = 1.23) was lower than in other locations in the Gran Chaco without vector control [31, 41, 69, 70]. Such force of infection implies that every year nearly 34 new human infections would have occurred in the study area if pre-intervention transmission conditions had remained unchanged.

Of the 122 children born after program onset and tested for antibodies to *T. cruzi*, the only seropositive child likely contracted the infection from her *T. cruzi*-seropositive mother who reported no history of etiological treatment. Similarly, a district-wide serosurvey across Pampa del Indio municipality in 2016 revealed no *T. cruzi*-seropositive child born after the onset of the intervention program except one attributed to transplacental transmission, and no incident cases were detected among residents who had been seronegative at baseline [43]. These serosurvey outcomes provide further support for the interruption of vector-borne transmission to humans across rural areas of Pampa del Indio [43].

The generalized linear mixed model identified several risk factors associated with children or all-ages *T. cruzi* infection. Duration of exposure to *T. infestans* was positively and strongly associated with *T. cruzi* infection, as reported by several studies [24, 41, 62]. The number of infected co-inhabitants had high RI in Model 1 and was also positively associated with human infection. This variable implies vector-borne transmission risk in two senses: it indicates the number of local parasite sources for triatomines and pinpoints the domicile as a high-risk place for vector-borne transmission in the past. Nevertheless, the number of infected co-inhabitants may not reflect the current risk status of a household since domestic infestation with *T. infestans* had been virtually suppressed after community-wide insecticide spraying. Residential stability, measured as duration of residence in a given house, is especially important for a population with frequent mobility as in this study. In the past, we detected that moving the household location was a shared feature among some Qom families (formerly semi-nomadic), and we called them “movers” [44]. Movers represented a fraction of the population exhibiting the highest vulnerability, which had a higher risk of residing in an infested house. They accounted for 14% of the population in Area III in a 3-year period. To address the occurrence of “movers” (i.e. detect transient changes in residence location) an ad hoc study design was conducted in Area III, which was not implemented in Area II and IV. Therefore, we could not classify the study households as “movers” or “non-movers.” We addressed this limitation by including residence time in the current house and the place of previous residence in univariate analyses. The relationship between domiciliary infestation and infection of children was modified by household mobility in Area III [44]; in non-infested houses, the infection risk in children was higher among movers than among households with stable residency, while the reverse was observed in infested houses [44]. In our dataset, people with previous residence in urban Pampa del Indio exhibited a lower risk of infection than people with stable residence in the study area. In contrast, people coming from other rural areas of Pampa del Indio municipality exhibited a higher risk of infection. People with previous residence outside Pampa del Indio showed a similar prevalence to stable residents, and no significant difference in the observed risk of infection was detected (Additional Information Table S4). These findings reveal the importance of incorporating variables that consider the history of exposure rather than a snapshot of present conditions, particularly in intervened scenarios like in this study in risk factor analysis.

The strong positive association between entomological indices and human *T. cruzi* infection has been verified across epidemiological settings [29, 30, 35, 41, 29–30]. In this study, the sole entomological variable that considered the individual’s lifetime exposure was self-reported inhabiting an infested house. This variable was critical in the case of residential infestations elsewhere [72–74], had the best performance among entomological predictors in the univariate analysis and was therefore included in the risk indices despite having a moderate RI in the GLMM. However, in the current study, the occurrence of triatomines by timed manual searches during baseline and surveillance period was not statistically associated with human infection, in contrast to previous reports [71, 75, 76]. As mentioned above, this observation is likely related to the fact that infestation data were available for the previous 6–9 years, whereas human infection probably occurred before that period, and that during the surveillance phase infestation mainly occurred in peridomestic sites associated with chickens [22]. In addition, the high mobility rate inside the municipality probably also affected the association between entomological indices and human infection as observed in Area III [44]. Immigration of *T. cruzi*-infected people could add to this discrepancy. However, only 9.2% of the tested individuals reportedly lived outside the municipality, including presumably non-infested urban areas, suggesting this potential source of *T. cruzi*-infected people was negligible in this population.

Indigenous people usually experienced higher risks of infection for diverse NTDs [77]. Several indigenous groups exhibited a higher prevalence of *T. cruzi* infection than creoles inhabiting the same area [40, 61, 65, 78, 79]. In this study, we observed strong heterogeneities between and within ethnic groups. Ethnic background was correlated with some socioeconomic variables such as the goat equivalent index, educational climate, time of residence, inhabiting a house with suitable walls for triatomines and critical overcrowding. Qom households exhibited worse socioeconomic conditions than creoles. However, creoles from Area IV exhibited the highest seroprevalence and force of infection found in this study, whereas creoles from Area II displayed the lowest levels and Qom people from either area had intermediate ones. This may be the first report of creoles showing higher prevalence of *T. cruzi* infection than an indigenous group residing in the same rural area. Creoles from Area IV inhabited remote rural sections with difficult accessibility (TCZ, CQ and EP) and had the lowest goat-equivalent indices of all creole households across the municipality [37, 42, 45]. Nevertheless, on average across the four areas, creole households exhibited higher goat-equivalent indices than Qom ones. These results point to a complex set of risk determinants, as in Area III [44], which escapes from the oversimplifying notion of equaling household ethnicity to infection risk as supported by the low RIs retrieved for the simple effects of ethnicity, area and their interaction when adjusted for other variables.

Regarding non-vectorial transmission routes, maternal serostatus displayed high RI for *T. cruzi* infection among children born before program onset. Maternal serostatus informs about the possibility of transplacental transmission and the number of infected household residents. According to a recent report on the situation of Chagas disease in the Americas [80], transplacental transmission has gained relative relevance in the context of more widespread vector control, in accordance with our findings among children born after program onset. Our preliminary estimate of the probability of vertical transmission (2%) is similar to the ones recorded in other regions [81].]. Oral transmission appears to be negligible in our study area and elsewhere in Argentina, where no oral outbreak of human *T. cruzi* infection has been reported so far [8].

The developed risk indices exhibited promising results identifying 93.9–98.1% of households with *T. cruzi*-seropositive children based on three questions easy to deliver in the field. They also have some limitations; one is the lack of validation with an external data set. Therefore, the reported sensitivity and specificity must be considered cautiously. The inclusion of “self-reported inhabiting an infested house” increased the frequency of false-positive cases. The low specificity implies more extended field operations. As the number of children inhabiting infested houses is expected to drop with control intervention progress, the incorrectly identified households will also decline. The presence of cracked walls (suitable walls for triatomines), easy to canvass during house-to-house inspections for triatomines has been routinely registered by vector control programs at least in Argentina. As a proxy of triatomine infestation risk [42], the inclusion of suitable walls for triatomines in other endemic regions may need to be tailored to the local triatomine species. Another limitation of the second index is its dependence on serological data. However, this constraint may be negligible in areas where serological data are accessible; for example, they could be obtained from the mandatory Chagas disease serodiagnosis of pregnant women in Argentina and in other countries with similar legislation [82].

Different approaches have been proposed for identifying infected children in endemic areas. Researchers were able to identify individuals with increased risk of infection by using indices based on the number of infected co-inhabitants or entomological data [33, 34]. Recent studies aimed to identify households with infected children through extensive data analysis though this approach may have little applicability in routine operations [28]. Here, our aim was to keep the indices simple to use, with the selected questions easy to answer by residents. For the index development, we chose the stratification at the household rather than person level since it is simpler during fieldwork to search a house than an individual. Household clustering of human infection further supports this approach [35].

We identified three local hotspots of infection of children close to hotspots of triatomine abundance [22, 36, 37]. One was associated with houses not inspected at baseline and with the occurrence of pyrethroid-resistant *T. infestans* [22, 37]. The observed spatial overlap between hotspots supports that children’s infections were mainly vector-borne and occurred before the onset of control efforts. A study conducted by Levy et al. [35] suggested the combined use of spatial analysis and risk indices to identify households harboring infected children in a low-prevalence urban setting, thus saving labor-related costs at the expense of other costs related to data analysis. Further studies are needed to validate these approaches.

The data available for our analyses has some limitations. We were unable to include some variables of interest, such as baseline house infestation and triatomine infection status [30, 35, 41, 70, 75], because of the systematic sampling of houses at baseline in Area II [37]. Self-reported lifetime residence in an infested house [71] is expected to suffer from recall bias. Maternal serostatus was another important variable with missing data for older people, as was the frequency of infected adult people; hence, it could not be included in the regression analysis of the complete dataset.

Our serosurvey achieved a fairly high diagnostic coverage (~ 50%) of the study population, especially because only 13% of the individuals reported had a previous serodiagnosis, showing the feasibility of achieving high coverage in rural endemic zones [17]. There is a chance for non-participation bias. This might happen if the non-participating fraction of people does not represent a random sample of the population or if the reason for non-participating was related to a previous known serostatus for *T. cruzi*. This is unlikely since < 14% of the participants reportedly had a previous serodiagnosis. An additional source of bias could happen if a whole age class was excluded from the analysis. We addressed this potential issue by conducting house-to-house visits to reach all rural inhabitants. As the coverage of children under 18 years of age was 55.4%, we estimated that 513 children may have remained without access to serodiagnosis, and 71 (95% CI = 57–88) likely were seropositive for *T. cruzi*. One of the strengths of this study was the consistent use of serological tests and vector detection methods across areas and periods for almost 10 years, which allowed direct comparisons across time and space [17, 38, 39, 43, 83].

The proposed indices may be suitable for other endemic situations in which the main triatomine vector is domiciliated and the local inhabitants report exposure to triatomines (i.e. they need to recognize the vector species). Given that the indices rely on self-reported exposure to triatomines, migration background is not expected to alter index validity. Likewise, these indices are independent of vector control history. However, in the scenario of massive exposure to triatomines, the discriminatory power of the indices will drop abruptly since almost all households will be marked as risky. Although the serodiagnosis of *T. cruzi* infection at the age of 6 years is guaranteed by a National Law, it has not been implemented across all rural endemic areas. Therefore, developed indices may assist public health officials working with limited resources to prioritize households with higher odds of harboring infected children.

The seroprevalence of children born after the onset of interventions and house infestation data for Areas II and IV of Pampa del Indio support the interruption of vector-borne transmission of *T. cruzi* to humans 10 years after program onset [22, 36, 43]. This status offers room to address new challenges such as detection of transplacental transmission and ensuring a wider access to serodiagnosis and treatment of rural residents, especially of seropositive women of childbearing age. The risk indices here presented may help to scale up such interventions in a cost-effective fashion.

## Conclusions

Nearly a quarter of the study population was seropositive for *T. cruzi* infection 6–9 years after the onset of sustained vector control interventions in a highly endemic municipality of the Argentine Chaco. We revealed strong heterogeneities in the infection risk between and within ethnic groups inhabiting two neighboring rural areas. Creole households from Area IV exhibited a higher risk of infection than Qom households of either area. Two simple risk indices identified nearly all the households harboring *T. cruzi*-seropositive children at the expense of low specificity. These indices may be easily implemented and scaled up across endemic areas to improve the access of rural residents to diagnosis and treatment of *T. cruzi* infection in pursuit of Sustainable Development Goals and disease prevention. Vector control actions should be sustained over time to prevent renewed vector-borne transmission.

### Supplementary Information

**Additional file 1: Table S1.** STROBE checklist.

**Additional file 2: Table S2.** Household socio-demographic variables for Area II and IV as of 2016 (unless otherwise indicated) and house infestation with *Triatoma infestans*, Pampa del Indio, Chaco. The number of households is indicated between parentheses.

**Additional file 3: Table S3.** Mean age (in years), number and percentage (between brackets) of demographic characteristics for the complete and tested populations from Areas II and IV by ethnic group, Pampa del Indio, Chaco, 2016.

**Additional file 4: Text 1 and Table S4. **Potential risk factors for *Trypanosoma cruzi* infection in residents of Areas II and IV of Pampa del Indio, Chaco, 2014–2017. *N*: number of individuals with data, *OR* univariate odds ratio, *95% CI* 95% confidence interval, *NA* data not available.

**Additional file 5: Figure S1.** Global spatial analysis of the household number of *Trypanosoma cruzi*-seropositive children ≤18 years of age at baseline. The grey area is the 95% confidence envelope of the mean L.

**Additional file 6: Figure S1.** Database.

## Data Availability

The dataset supporting the conclusions of this article is included within the article and in the supplementary material.

## Abbreviations

AUC: Area under the ROC curve
CI: Confidence interval
CM: Colonia Mixta, Area IV
CQ: Campo Cacique, Area IV
CY: Campo Alemany, Area IV
Df: Degrees of freedom
ELISA: Enzyme-linked immunosorbent assays
EP: Ex-Parque, Area IV
GLMM: Generalized linear mixed-effects models
H2: La Herradura, Area IV
LA: Cancha Larga, Area II
LC: Lote Cuatro, Area II
LM: Las Muñecas, Area IV
ME: Campo Medina, Area II
NTD: Neglected Tropical Disease
NU: Campo Nuevo, Area II
OM: Pampa Ombú, Area II
RI: Relative importance
ROC: Receiver-operating characteristic curve
TCZ: Tacuruzal, Area IV
FOI: Mean annual force of infection

## References

[1] Hotez PJ, Bottazzi ME, Franco-Paredes C, Ault SK, Periago MR (2008). The neglected tropical diseases of Latin America and the Caribbean: a review of disease burden and distribution and a roadmap for control and elimination. PLoS Negl Trop Dis.

[2] Ault SK (2007). Chagas Disease and neglected diseases: changing poverty and exclusion.

[3] WHO (2020). Ending the neglect to attain the sustainable development goals: a road map for neglected tropical diseases 2021–2030.

[4] Hotez PJ, Alvarado M, Basáñez M-G, Bolliger I, Bourne R, Boussinesq M (2014). The global burden of disease study 2010: interpretation and implications for the neglected tropical diseases. PLoS Negl Trop Dis.

[5] World Health Organization (2002). Control of Chagas Disease : second report of the WHO expert committee.

[6] Howard EJ, Xiong X, Carlier Y, Sosa Estani SA, Buekens P (2014). Frequency of the congenital transmission of *Trypanosoma cruzi*: a systematic review and meta-analysis. BJOG Int J Obstet Gynaecol.

[7] de Góes CE, Dos Santos SO, Sojo-Milano M, Amador ECC, Tatto E, Souza DSM (2017). Acute Chagas Disease in the Brazilian Amazon: epidemiological and clinical features. Int J Cardiol.

[8] López-García A, Gilabert JA (2023). Oral transmission of Chagas disease from a one health approach: a systematic review. Trop Med Int Health.

[9] Wendel S (2010). Transfusion transmitted Chagas Disease : is it really under control?. Acta Trop.

[10] Dias J, Silveira AC, Schofield CJ (2002). The impact of Chagas Disease control in Latin America—a review. Mem Inst Oswaldo Cruz.

[11] Gorla DE, Hashimoto K, Telleria J, Tibayrenc M (2017). Control strategies against Triatominae. Am Trypanos Chagas Dis.

[12] Gürtler RE, Cecere MC, Guarneri A, Lorenzo M (2021). Chagas Disease vector control. Triatominae - Biol Chagas Dis Vectors.

[13] Schofield CJ, Jannin J, Salvatella R (2006). The future of Chagas disease control. Trends Parasitol.

[14] Mougabure-Cueto G, Picollo MI, Guarneri A, Lorenzo M (2021). Insecticide Resistance in Triatomines. Triatominae—biol Chagas disease vectors.

[15] Organización Panamericana de la Salud (2019). Control, interrupción de la transmisión y eliminación de la enfermedad de Chagas como problema de salud pública. Guía de evaluación, verificación y validación.

[16] Carlier Y, Altcheh J, Angheben A, Freilij H, Luquetti AO, Schijman AG (2019). Congenital Chagas Disease: updated recommendations for prevention, diagnosis, treatment, and follow-up of newborns and siblings, girls, women of childbearing age, and pregnant women. PLoS Negl Trop Dis.

[17] Sartor P, Colaianni I, Cardinal MV, Bua J, Freilij H, Gürtler RE (2017). Improving access to Chagas Disease diagnosis and etiologic treatment in remote rural communities of the Argentine Chaco through strengthened primary health care and broad social participation. PLoS Negl Trop Dis.

[18] Martínez-Parra AG, Pinilla-Alfonso MY, Abadía-Barrero CE (2018). Sociocultural dynamics that influence Chagas Disease health care in Colombia. Soc Sci Med.

[19] Rey León JA (2020). La justicia social en salud y su relación con la enfermedad de Chagas.

[20] Yun O, Lima MA, Ellman T, Chambi W, Castillo S, Flevaud L (2009). Feasibility, drug safety, and effectiveness of etiological treatment programs for Chagas disease in Honduras, Guatemala, and Bolivia: 10-year experience of Médecins Sans Frontières. PLoS Negl Trop Dis.

[21] Alonso-Padilla J, Cortés-Serra N, Pinazo MJ, Bottazzi ME, Abril M, Barreira F (2019). Strategies to enhance access to diagnosis and treatment for Chagas disease patients in Latin America. Expert Rev Anti Infect Ther.

[22] Fernández MP, Gaspe MS, Sartor P, Gürtler RE. Human *Trypanosoma cruzi* infection risk is driven by eco-social interactions in rural communities of the Argentine Chaco. PLoS Negl Trop Dis. 2019;13:e0007430 10.1371/journal.pntd.0007430 PMC6936860 31841558

[23] Gürtler RE, Gaspe MS, Macchiaverna NP, Enriquez GF, Rodríguez-Planes LI, del Fernández MP (2023). The Pampa del Indio project: district-wide quasi-elimination of *Triatoma infestans* after a 9-year intervention program in the Argentine Chaco. PLoS Negl Trop Dis.

[24] Basombrío MA, Segovia A, Peralta Ramos M, Esteban E, Stumpf R, Jurgensen P (1999). Endemic *Trypanosoma cruzi*, infection in indian population of the Gran Chaco territory of South America: performance of diagnostic assays and epidemiological features. Ann Trop Med Parasitol.

[25] Black CL, Ocaña S, Riner D, Costales JA, Lascano MS, Davila S (2007). Household risk factors for *Trypanosoma cruzi* seropositivity in two geographic regions of Ecuador. J Parasitol.

[26] Cucunubá ZM, Flórez AC, Cárdenas Á, Pavía P, Montilla M, Aldana R (2012). Prevalence and risk factors for Chagas disease in pregnant women in Casanare. Colombia Am J Trop Med Hyg.

[27] Grijalva MJ, Escalante L, Paredes RA, Costales JA, Padilla A, Rowland EC (2003). Seroprevalence and risk factors for *Trypanosoma cruzi* infection in the Amazon region of Ecuador. Am J Trop Med Hyg.

[28] Levy MZ, Bowman NM, Kawai V, Plotkin JB, Waller LA, Cabrera L (2009). Spatial patterns in discordant diagnostic test results for Chagas disease: links to transmission hotspots. Clin Infect Dis.

[29] Mott KE, de Oliveira TS, Sherlock I, Morrow RH, Hoff R, Muniz TM (1978). House construction, triatomine distribution, and household distribution of seroreactivity to *Trypanosoma cruzi* in a rural community in Northeast Brazil. Am J Trop Med Hyg.

[30] Piesman J, Weller TH, Hoff R, Mota E, Todd CW, Sherlock IA (1985). Association between household triatomine density and incidence of *Trypanosoma cruzi* infection during a nine-year study in Castro Alves, Bahia. Brazil Am J Trop Med Hyg.

[31] Samuels AM, Clark EH, Galdos-Cardenas G, Wiegand RE, Ferrufino L, Menacho S (2013). Epidemiology of and impact of insecticide spraying on Chagas disease in communities in the Bolivian Chaco. PLoS Negl Trop Dis.

[32] Gürtler RE, del Fernández MP, Cardinal MV, Guarneri A, Lorenzo M (2021). Eco-epidemiology of the domestic vector-borne transmission of *Trypanosoma cruzi*. Triatominae biol Chagas disease vectors.

[33] Mott KE, Lehman JS, Hoff R, Morrow RH, Muniz TM, Sherlock IA (1976). The epidemiology and household distribution of seroreactivity to *Trypanosoma cruzi* in a rural community in Northeast Brazil. Am J Trop Med Hyg.

[34] de Andrade ALSS, Zicker F, Garcia da Silva I, Souza JMP, Martelli CMT (1995). Risk factors for *Trypanosoma cruzi* infection among children in Central Brazil: a case-control study in vector control settings. Am J Trop Med Hyg.

[35] Levy MZ, Kawai V, Bowman NM, Waller LA, Cabrera L, Pinedo-Cancino VV (2007). Targeted screening strategies to detect *Trypanosoma cruzi* infection in children. PLoS Negl Trop Dis.

[36] Gürtler RE, Enriquez GF, Gaspe MS, Macchiaverna NP, del Fernández MP, Rodríguez-Planes LI (2023). The Pampa del Indio project: sustainable vector control and long-term declines in the prevalence and abundance of *Triatoma infestans* infected with *Trypanosoma cruzi* in the Argentine Chaco. Parasit Vectors.

[37] Provecho YM, Gaspe MS, del Fernández MP, Gürtler RE, Byrd J (2017). House reinfestation with *Triatoma infestans* (Hemiptera: Reduviidae) after community-wide spraying with insecticides in the Argentine Chaco: a multifactorial process. J Med Entomol.

[38] Gurevitz JM, Gaspe MS, Enriquez GF, Provecho YM, Kitron U, Gürtler RE (2013). Intensified surveillance and insecticide-based control of the Chagas disease vector *Triatoma infestans* in the Argentinean Chaco. PLoS Negl Trop Dis.

[39] Gaspe MS, Provecho YM, del Fernández MP, Vassena CV, Santo-Orihuela PL, Gürtler RE (2018). Beating the odds: sustained Chagas disease vector control in remote indigenous communities of the Argentine Chaco over a seven-year period. PLoS Negl Trop Dis.

[40] Cardinal MV, Orozco MM, Enriquez GF, Ceballos LA, Gaspe MS, Alvarado-Otegui JA (2014). Heterogeneities in the ecoepidemiology of *Trypanosoma cruzi* infection in rural communities of the Argentinean Chaco. Am J Trop Med Hyg.

[41] Cardinal MV, Sartor PA, Gaspe MS, Enriquez GF, Colaianni I, Gürtler RE (2018). High levels of human infection with *Trypanosoma cruzi* associated with the domestic density of infected vectors and hosts in a rural area of northeastern Argentina. Parasit Vectors.

[42] Gurevitz JM, Ceballos LA, Gaspe MS, Alvarado-Otegui JA, Enriquez GF, Kitron U (2011). Factors affecting infestation by *Triatoma infestans* in a rural area of the humid Chaco in Argentina: a multi-model inference approach. PLoS Negl Trop Dis.

[43] Cardinal MV, Enriquez GF, Macchiaverna NP, Argibay HD, del Fernández MP, Alvedro A (2021). Long-term impact of a ten-year intervention program on human and canine *Trypanosoma cruzi* infection in the Argentine Chaco. PLoS Negl Trop Dis.

[44] del Fernández MP, Gaspe MS, Gürtler RE (2019). Inequalities in the social determinants of health and Chagas Disease transmission risk in indigenous and creole households in the Argentine Chaco. Parasit Vectors.

[45] Gaspe MS, Provecho YM, Cardinal MV, del Fernández MP, Gürtler RE (2015). Ecological and sociodemographic determinants of house infestation by *Triatoma infestans* in indigenous communities of the Argentine Chaco. PLoS Negl Trop Dis.

[46] INDEC (2010). Censo nacional de población, hogares y vivienda.

[47] Krebs CJ (1999). Ecological methodology.

[48] Brown LD, Cai TT, DasGupta A (2001). Interval estimation for a binomial proportion. Stat Sci.

[49] R Core Team (2020). R: a language and environment for statistical computing.

[50] Bates D, Mächler M, Bolker B, Walker S (2015). Fitting linear mixed-effects models using lme4. J Stat Softw.

[51] Barton K. MuMIn: multi-model inference. R package version 1.40. 4. https://cran.r-project.org/package=MuMIn. 2016.

[52] Lele SR, Keim JL, Solymos P. Resource selection: resource selection (probability) functions for use-availability data. https://cran.r-project.org/package=ResourceSelection. 2014.

[53] Fox J, Weisberg S (2011). An R Companion to Applied Regression.

[54] Anagnostopoulos C, Hand DJ. hmeasure: the H-measure and other scalar classification performance metrics. R Package Version 10–2. https://cran.r-project.org/package=hmeasure. 2019.

[55] Muench H (2013). Catalytic models in epidemiology.

[56] Wiegand T, Moloney KA (2013). Handbook of spatial point-pattern analysis in ecology.

[57] Getis A, Ord JK (1995). Local spatial autocorrelation statistics: distributional issues and an application. Geogr Anal.

[58] Baddeley A, Rubak E, Turner R (2015). Spatial point patterns: methodology and applications.

[59] Bivand RS, Wong DWS (2018). Comparing implementations of global and local indicators of spatial association. TEST.

[60] Alonso JM, Fabre AR, Galván M, Lucero RH, Brusés BL, Kuc A (2009). La Enfermedad de Chagas en poblaciones aborígenes del Noreste de Argentina. Enfermedades Emerg.

[61] Biancardi MA, Conca Moreno M, Torres N, Pepe C, Altcheh J, Freilij H (2003). Seroprevalence of Chagas disease in 17 rural communities of “Monte Impenetrable”. Chaco Province Med B Aires.

[62] Chippaux J-P, Postigo JR, Santalla J, Schneider D, Brutus L (2008). Epidemiological evaluation of Chagas Disease in a rural area of southern Bolivia. Trans R Soc Trop Med Hyg.

[63] Diosque P, Padilla AM, Cimino RO, Marino Cardozo R, Sanchez Negrette O, Marco JD (2004). Chagas Disease in rural areas of Chaco Province, Argentina: epidemiologic survey in humans, reservoirs, and vectors. Am J Trop Med Hyg.

[64] Gürtler RE, Chuit R, Cecere MC, Castañera MB, Cohen JE, Segura EL (1998). Household prevalence of seropositivity for *Trypanosoma cruzi* in three rural villages in northwest Argentina: environmental, demographic, and entomologic associations. Am J Trop Med Hyg.

[65] Moretti E, Castro I, Franceschi C, Basso B (2010). Chagas Disease: serological and electro cardiographic studies in Wichi and Creole communities of Misión Nueva Pompeya, Chaco. Argentina Mem Inst Oswaldo Cruz.

[66] Sosa Estani SA, Dri L, Touris C, Abalde S, Dell’Arciprete A, Braunstein J (2009). Transmisión vectorial y congénita del *Trypanosoma cruzi* en Las Lomitas. Formosa Med B Aires.

[67] Spinicci M, Gabrielli S, Rojo D, Gamboa H, Macchioni F, Mantella A (2020). Trypanosoma cruzi infection in the human population of the Bolivian Chaco: four serosurveys over a 26-year period (1987–2013). J Infect Dev Ctries.

[68] Fleitas PE, Floridia-Yapur N, Nieves EE, Echazu A, Vargas PA, Caro NR (2022). *Strongyloides stercoralis* and *Trypanosoma cruzi* coinfections in a highly endemic area in Argentina. PLoS Negl Trop Dis.

[69] Hopkins T, Gonçalves R, Mamani J, Courtenay O, Bern C (2019). Chagas disease in the Bolivian Chaco: persistent transmission indicated by childhood seroscreening study. Int J Infect Dis.

[70] Gürtler RE, Cecere MC, Lauricella MA, Petersen RM, Chuit R, Segura EL (2005). Incidence of *Trypanosoma cruzi* infection among children following domestic reinfestatoin after insecticide spraying in rural northwestern Argentina. Am J Trop Med Hyg.

[71] Bowman NM, Kawai V, Gilman RH, Bocangel C, Galdos-Cardenas G, Cabrera L (2011). Autonomic dysfunction and risk factors associated with *Trypanosoma cruzi* infection among children in Arequipa. Peru Am J Trop Med Hyg.

[72] Enriquez GF, Cecere MC, Alvarado-Otegui JA, Alvedro A, Gaspe MS, Laiño MA (2020). Improved detection of house infestations with triatomines using sticky traps: a paired-comparison trial in the Argentine Chaco. Parasit Vectors.

[73] Abad-Franch F, Vega MC, Rolón MS, Santos WS, de Arias RA (2011). Community participation in Chagas disease vector surveillance: systematic review. PLoS Negl Trop Dis.

[74] Dumonteil E, Ramirez-Sierra MJ, Ferral J, Euan-Garcia M, Chavez-Nuñez L (2009). Usefulness of community participation for the fine temporal monitoring of house infestation by non-domiciliated triatomines. J Parasitol.

[75] Alroy KA, Huang C, Gilman RH, Quispe Machaca VR, Marks MA, Ancca Juárez J (2015). Prevalence and transmission of *Trypanosoma cruzi* in people of rural communities of the high jungle of Northern Peru. PLoS Negl Trop Dis.

[76] Delgado S, Castillo Neyra R, Quispe Machaca VR, Ancca Juárez J, Chou Chu L, Verastegui MR (2011). A history of Chagas Disease transmission, control, and re-emergence in peri-rural La Joya. Peru PLoS Negl Trop Dis.

[77] Hotez PJ (2014). Aboriginal populations and their neglected tropical diseases. PLoS Negl Trop Dis.

[78] Cucunubá ZM, Nouvellet P, Peterson JK, Bartsch SM, Lee BY, Dobson AP (2018). Complementary paths to Chagas Disease elimination: the impact of combining vector control with etiological treatment. Clin Infect Dis.

[79] Lucero RH, Brusés BL, Cura CI, Formichelli LB, Juiz N, Fernández GJ (2016). Chagas Disease in aboriginal and creole communities from the Gran Chaco Region of Argentina: seroprevalence and molecular parasitological characterization. Infect Genet Evol.

[80] PAHO (2018). Enfermedad de Chagas en las Américas: una revisión de la situación actual de salud pública y su visión para el futuro.

[81] Santana KH, Oliveira LGR, Barros de Castro D, Pereira M (2020). Epidemiology of Chagas Disease in pregnant women and congenital transmission of Trypanosoma cruzi in the Americas: systematic review and meta-analysis. Trop Med Int Health.

[82] Balestrini A, Pampuro JJB, Hidalgo E, Estrada JH (2007). Ley 26.281 de Prevención y control del Chagas.

[83] Provecho YM, Gaspe MS, del Fernández MP, Enriquez GF, Weinberg D, Gürtler RE (2014). The peri-urban interface and house infestation with *Triatoma infestans* in the Argentine Chaco: an underreported process?. Mem Inst Oswaldo Cruz.

