# Supplementary material for: Human Trypanosoma cruzi infection in the Argentinean Chaco: risk factors and identification of households with infected children for treatment
Source: Parasit Vectors. 2024 Jan 29;17:41. doi: 10.1186/s13071-024-06125-8 (PMC10826042; doi:10.1186/s13071-024-06125-8)
Supplement: Supplementary file 2 — Additional file 2: Table S2. Household socio-demographic variables for Area II and IV as of 2016 (unless otherwise indicated) and house infestation with Triatoma infestans, Pampa del Indio, Chaco. The number of households is indicated between parentheses. [file 13071_2024_6125_MOESM2_ESM.docx]

Additional file 2: Table S2: Household socio-demographic variables for Area II and IV as of 2016 (unless otherwise indicated) and house infestation with *Triatoma infestans*, Pampa del Indio, Chaco. The number of households is indicated between parentheses.

|  | Area II | | | Area IV | | |
| --- | --- | --- | --- | --- | --- | --- |
| Variable | Qom | Creole | Total | Qom | Creole | Total |
| % inhabited houses | 70.9 | 29.1 | 100 (409) | 34.4 | 64.8 | 100 (250) |
| % infested house at baseline ^a^ | 14.9 (94) | 14.0 (86) | 14.4 (180) | 28.2 (71) | 14.2 (183) | 18.1 (254) |
| % infested houses as of 2016 | 0 (228) | 0 (93) | 0 (327) | 2.4 (82) | 2.1 (143) | 1.8 (228) |
| % households with children <15 years of age | 66.8 (232) | 38.8(85) | 59.3 (317) | 78.4 (74) | 38.3 (133) | 52.7 (207) |
| % houses with suitable walls for triatomines | 63.9 (208) | 38.4 (86) | 56.8 (336) | 58.0 (69) | 33.6 (137) | 41.2 (228) |
| Mean no. of peridomestic sites ± SD^b^ | 1.9 ± 1.8 (259) | 3.3 ± 2.8 (136) | 2.3 ± 2.3 (395) | 0.8 ± 1.1 (63) | 2.5 ± 2.0 (118) | 1.9 ± 1.9 (184) |
| Mean no. of residents per household ± SD | 5.0 ± 2.9 (290) | 3.3 ± 2.1 (119) | 4.5 ± 2.8 (409) | 5.3 ± 2.8 (86) | 3.4 ± 2.7 (162) | 4.1 ± 2.9 (250) |
| Median goat-equivalent (Q1-Q3) | 1.0 (0.2-7.1) (208) | 26.8 (1.6-94.1) (83) | 1.5 (0.2-15.1) (328) | 0.8 (0.2-3.5) (69) | 9.7 (0.7-65.7) (138) | 1.5 (0.2-21.8) (229) |
| Household educational level ± SD^c^ | 4.5 ± 3.1 (198) | 5.5 ± 2.9 (58) | 4.7 ± 3.1 (256) | 5.1 ± 2.1 (56) | 5.7 ± 3.5 (69) | 5.4 ± 2.9 (125) |
| Mean residence time (in years) ± SD^d^ | 18.6 ± 22.4 (289) | 22.5 ± 19.9 (119) | 19.7 ± 2179 (409) | 8.1 ± 11.6 (84) | 16.8 ± 18.4 (160) | 13.6 ± 16.8 (248) |
| % households with critical overcrowding ^e^ | 64.6 (147) | 26.1 (69) | 52.3 (216) | 63.3 (60) | 23.1 (117) | 36.5 (178) |
| % households using insecticide ^f^ | 40.2 (254) | 61.8 (136) | 47.7 (390) | 77.8 (27) | 80.7 (140) | 80.2 (167) |

Year when the variable was registered: ^a^ II: 2008, IV: 2009-2010; ^b^ II: 2009, IV: 2015, ^c^ during serosurveys; ^d^ II: 2011, IV: 2013; ^e^ II: 2011, IV: 2015; ^f^ II: 2012, IV: 2013
