# Supplementary material for: Human Trypanosoma cruzi infection in the Argentinean Chaco: risk factors and identification of households with infected children for treatment
Source: Parasit Vectors. 2024 Jan 29;17:41. doi: 10.1186/s13071-024-06125-8 (PMC10826042; doi:10.1186/s13071-024-06125-8)
Supplement: Supplementary file 3 — Additional file 3: Table S3. Mean age (in years), number and percentage (between brackets) of demographic characteristics for the complete and tested populations from Areas II and IV by ethnic group, Pampa del Indio, Chaco, 2016. [file 13071_2024_6125_MOESM3_ESM.docx]

Additional file 3: Table S3: Mean age (in years), number and percentage (between brackets) of demographic characteristics for the complete and tested populations from Areas II and IV by ethnic group, Pampa del Indio, Chaco, 2016.

|  | Area II | | | | | | Area IV | | | | | |
| --- | --- | --- | --- | --- | --- | --- | --- | --- | --- | --- | --- | --- |
|  | Census (%) | | | Examined (%) | | | Census (%) | | | Examined (%) | | |
| Ethnic group | Qom | Creole | Total | Qom | Creole | Total | Qom | Creole | Total | Qom | Creole | Total |
| Inhabitants | 1452 (77.8) | 414 (22.2) | 1866 (66.8) | 775 (81.7) | 174 (18.3) | 949 (50.9) | 379 (40.9) | 548 (59.1) | 927 (33.2) | 192 (49.5) | 196 (50.5) | 388 (41.9) |
| Females | 661 (45.5) | 186 (44.9) | 847 (45.4) | 390 (50.3) | 82 (47.1) | 472 (49.7) | 167 (44.1) | 241 (44.0) | 408 (44.0) | 88 (45.8) | 103 (52.6) | 191 (49.2) |
| No. of people ≤18 years of age | 670 (46.1) | 118 (28.5) | 788 (42.2) | 400 (51.6) | 55 (31.6) | 455 (47.9) | 158 (41.7) | 194 (35.4) | 352 (38.0) | 83 (43.2) | 89 (45.4) | 172 (44.3) |
| No. of children born after the onset of interventions | 220 (15.2) | 38 (9.2) | 258 (13.8) | 79 (10.2) | 19 (10.9) | 98 (10.3) | 65 (17.2) | 71 (13.0) | 136 (14.7) | 15 (7.8) | 9 (4.6) | 24 (6.2) |
| Mean age ± SD (in years) | 24.9 ± 18.5 | 35.6 ± 22.4 | 27.2 ± 19.9 | 23.8 ± 17.1 | 33.3 ± 22 | 25.5 ± 18.5 | 23.0 ± 18.3 | 31.1 ± 22.6 | 27.9 ± 21.4 | 25.9 ± 17.5 | 26.4 ± 19.7 | 26.1 ± 18.6 |
