# Supplementary material for: Human Trypanosoma cruzi infection in the Argentinean Chaco: risk factors and identification of households with infected children for treatment
Source: Parasit Vectors. 2024 Jan 29;17:41. doi: 10.1186/s13071-024-06125-8 (PMC10826042; doi:10.1186/s13071-024-06125-8)
Supplement: Supplementary file 4 — Additional file 4: Text 1 and Table S4. Potential risk factors for Trypanosoma cruzi infection in residents of Areas II and IV of Pampa del Indio, Chaco, 2014–2017. N: number of individuals with data, OR univariate odds ratio, 95% CI 95% confidence interval, NA data not available. [file 13071_2024_6125_MOESM4_ESM.docx]

Additional file 4: Text 1 and Table S4

Text 1: In univariate analysis, duration of exposure and the household number of infected co-inhabitants were strongly and positively associated with human infection with *T. cruzi* as well as maternal seropositivity and self-reported blood transfusion. Among vector-related variables, self-reported inhabiting an infested house and the abundance of domestic *T. infestans* at baseline were positively associated with human infection. Residing in a house with suitable walls for triatomines or having resided in another house from the same village or in other rural areas within the municipality were positively associated with *T. cruzi* infection. In contrast, being born after the onset of the intervention program, living in a household with high educational climate, and inhabiting the current house for 3-10 years were negatively associated with infection. No significant association was found with gender, the goat-equivalent index, the presence of a suitable roof for triatomines, and critical overcrowding. Similar outcomes were observed for children under 18 years of age born before interventions (a subset of the former dataset) except for educational climate and the relative abundance of *T. infestans*, which were not significantly associated with child infection (Table S4).

**Table S4: Potential risk factors for *T. cruzi* infection in residents of Areas II and IV of Pampa del Indio, Chaco, 2014-2017. N: number of individuals with data, OR: Univariate odds ratio; 95% CI: 95% confidence interval; *P*: p-value; NA: data not available.**

| Variable | Total population | | | | Children ≤18 years born before the onset of interventions | | | |
| --- | --- | --- | --- | --- | --- | --- | --- | --- |
|  | N | % infected | OR  (95% CI) | *P* | N | % infected | OR  (95% CI) | *P* |
| Duration of exposure (years) | 1337 | 24.8 | 1.04  (1.03-1.04) | <0.001 | 676 | 16.3 | 1.16  (1.11-1.21) | <0.001 |
| Born after program onset | | | | | - | - | - | - |
| No | 1215 | 27.2 | 1 |  | - | - | - | - |
| Yes | 122 | 0.8 | 0.02  (0.00-0.10) | <0.001 | - | - | - | - |
| Gender |  |  |  |  |  |  |  |  |
| Female | 663 | 24.6 | 1 |  | 345 | 17.4 | 1 |  |
| Male | 674 | 25.1 | 1.03  (0.80-1.32) | 0.84 | 331 | 15.1 | 0.85  (0.56-1.27) | 0.42 |
| Ethnicity |  |  |  |  |  |  |  |  |
| Creole | 370 | 26.5 | 1 |  | 166 | 19.9 | 1 |  |
| Qom | 967 | 24.2 | 0.89  (0.68-1.17) | 0.4 | 510 | 15.1 | 0.72  (0.46-1.14) | 0.14 |
| Area |  |  |  |  |  |  |  |  |
| II | 949 | 23.5 | 1 |  | 483 | 15.3 | 1 |  |
| IV | 388 | 28.1 | 1.27  (0.97-1.66) | 0.08 | 193 | 18.7 | 1.27  (0.81-1.95) | 0.29 |
| Number of seropositive co-inhabitants | | | |  |  |  |  |  |
| 0 | 596 | 22.0 | 1 |  | 261 | 9.2 | 1 |  |
| 1-2 | 570 | 21.1 | 0.95  (0.72-1.25) | 0.70 | 317 | 13.3 | 1.51  (0.89-2.60) | 0.13 |
| 3 or more | 171 | 47.4 | 3.19  (2.23-4.57) | <0.001 | 98 | 44.9 | 8.05  (4.56-14.54) | <0.001 |
| Maternal seropositivity | | |  |  |  |  |  |  |
| No | 570 | 16.0 | 1 |  | 344 | 10.5 | 1 |  |
| Yes | 341 | 24.0 | 1.67  (1.19-2.33) | 0.003 | 223 | 23.8 | 2.67  (1.69-4.26) | <0.001 |
| NA | 426 | 37.3 | - | - | 109 | 19.3 | - | - |
| Self-reported blood transfusion | | | | |  |  |  |  |
| No | 1298 | 24.0 | 1 |  | 668 | 15.9 | 1 |  |
| Yes | 39 | 51.3 | 3.33  (1.75-6.35) | <0.001 | 8 | 50.0 | 5.30  (1.24-22.74) | 0.02 |
| Presence of domestic *T. infestans* at baseline | | | | | |  |  |  |
| No | 1044 | 23.8 | 1 |  | 518 | 15.8 | 1 |  |
| Yes | 186 | 28.0 | 1.25  (0.87-1.76) | 0.22 | 101 | 12.9 | 0.78  (0.40-1.43) | 0.45 |
| NA | 107 | 29.9 | - | - | 57 | 26.3 | - | - |
| Abundance of domestic *T. infestans* at baseline | | | | |  |  |  |  |
|  | 604 | 25.0 | 1.03  (1.00-1.07) | 0.04 | 299 | 14.7 | 1.00  (0.93-1.05) | 0.94 |
| Self-reported inhabiting an infested house | | | | |  |  |  |  |
| No | 354 | 18.9 | 1 |  | 173 | 11.6 | 1 |  |
| Yes | 877 | 28.3 | 1.69  (1.25-2.3) | 0.001 | 432 | 19.4 | 1.85  (1.11-3.19) | 0.02 |
| NA | 106 | 16.0 | - | - | 71 | 8.5 | - | - |
| Household educational climate | | | |  |  |  |  |  |
| <6 | 788 | 26.6 | 1 |  | 370 | 17.8 | 1 |  |
| 6-10 | 459 | 22.4 | 0.80  (0.61 - 1.04) | 0.10 | 257 | 15.2 | 0.82  (0.53 - 1.26) | 0.38 |
| >10 | 32 | 9.4 | 0.28  (0.07 - 0.81) | 0.04 | 20 | 5.0 | 0.24  (0.01 - 1.20) | 0.17 |
| NA | 58 | 27.6 | - | - | 29 | 13.8 | - | - |
| Goat-equivalent index | | | |  |  |  |  |  |
| Q1= [0-0.04) | 314 | 25.8 | 1 |  | 161 | 18.0 | 1 |  |
| Q2= [0.04-0.26) | 318 | 22.0 | 0.81  (0.56 - 1.17) | 0.27 | 178 | 14.0 | 0.74  (0.41-1.33) | 0.32 |
| Q3= [0.26- 1.71) | 325 | 27.1 | 1.07  (0.75 - 1.52) | 0.71 | 161 | 18.0 | 1.00  (0.57-1.77) | 1.00 |
| Q4= [1.71-166.67) | 319 | 23.8 | 0.90  (0.63 - 1.29) | 0.57 | 151 | 13.9 | 0.74  (0.39-1.35) | 0.32 |
| NA | 61 | 27.9 | - | - | 25 | 24.0 | - | - |
| Time of residence | | |  |  |  |  |  |  |
| < 3 years | 109 | 33.0 | 1 |  | 70 | 30.0 | 1 |  |
| 3-10 years | 518 | 21.6 | 0.56  (0.36-0.88) | 0.01 | 266 | 14.3 | 0.39  (0.21-0.73) | 0.003 |
| > 10 years | 693 | 25.8 | 0.71  (0.46-1.10) | 0.12 | 338 | 15.1 | 0.41  (0.23-0.76) | 0.004 |
| NA | 17 | 29.4 | - | - | 2 | 0 | - | - |
| Presence of suitable roof for triatomines | | | |  |  |  |  |  |
| No | 955 | 25.4 | 1 |  | 481 | 16.6 | 1 |  |
| Yes | 277 | 21.3 | 0.79  (0.57-1.09) | 0.16 | 146 | 14.4 | 0.84  (0.49-1.39) | 0.52 |
| NA | 105 | 28.6 | - | - | 49 | 18.4 | - | - |
| Presence of suitable walls for triatomines | | | |  |  |  |  |  |
| No | 503 | 20.9 | 1 |  | 240 | 11.7 | 1 |  |
| Yes | 786 | 27.1 | 1.41  (1.08-1.84) | 0.01 | 418 | 18.9 | 1.76  (1.12-2.85) | 0.02 |
| NA | 48 | 29.2 | - | - | 18 | 16.7 | - | - |
| Past residence | | |  |  |  |  |  |  |
| Same village | 1100 | 23.5 | 1 |  | 602 | 15.8 | 1 |  |
| Same area | 30 | 43.3 | 2.48  (1.17-5.16) | 0.02 | 13 | 30.8 | 2.52  (0.72 - 7.55) | 0.14 |
| Another rural area of Pampa del Indio | 33 | 39.4 | 2.11  (1.01-4.26) | 0.04 | 9 | 55.6 | 6.50  (1.81 - 24.57) | 0.01 |
| Urban Pampa del Indio | 36 | 22.2 | 0.93  (0.39-1.97) | 0.85 | 16 | 0.0 | 0.16  (0.00 -1.21) | 0.09 |
| Outside Pampa del Indio | 123 | 24.4 | 1.05  (0.67-1.60) | 0.83 | 32 | 12.5 | 0.84  (0.26 - 2.12) | 0.73 |
| NA | 15 | 60.0 | - | - | 4 | 50.0 | - | - |
| Critical overcrowding | | |  |  |  |  |  |  |
| No | 322 | 21.7 | 1 |  | 133 | 12.0 | 1 |  |
| Yes | 575 | 26.6 | 1.31  (0.95-1.81) | 0.11 | 319 | 17.9 | 1.59  (0.90-2.97) | 0.13 |
| NA | 440 | 24.8 | - | - | 224 | 16.5 | - | - |
