# Supplementary material for: Human Trypanosoma cruzi infection in the Argentinean Chaco: risk factors and identification of households with infected children for treatment
Source: Parasit Vectors. 2024 Jan 29;17:41. doi: 10.1186/s13071-024-06125-8 (PMC10826042; doi:10.1186/s13071-024-06125-8)
Supplement: Supplementary file 5 — Additional file 5: Figure S1. Global spatial analysis of the household number of Trypanosoma cruzi-seropositive children ≤18 years of age at baseline. The grey area is the 95% confidence envelope of the mean L. [file 13071_2024_6125_MOESM5_ESM.docx]

Additional file 5: Figure S1: Global spatial analysis of the household number of *T. cruzi*-seropositive children ≤18 years of age at baseline. The grey area is the 95% confidence envelope of the mean L.

**
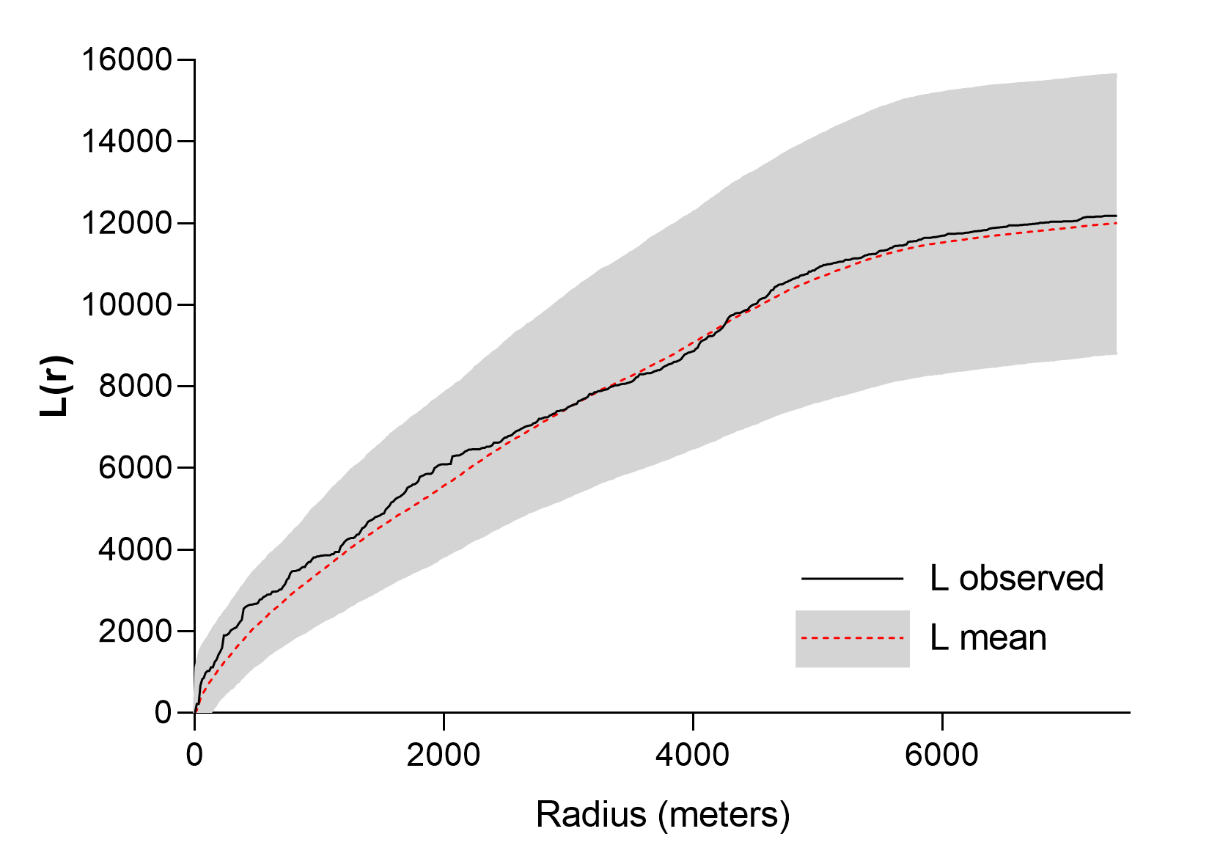
**
